# Genomic Targets and Features of BarA-UvrY (-SirA) Signal Transduction Systems

**DOI:** 10.1371/journal.pone.0145035

**Published:** 2015-12-16

**Authors:** Tesfalem R. Zere, Christopher A. Vakulskas, Yuanyuan Leng, Archana Pannuri, Anastasia H. Potts, Raquel Dias, Dongjie Tang, Bryan Kolaczkowski, Dimitris Georgellis, Brian M. M. Ahmer, Tony Romeo

**Affiliations:** 1 Department of Microbiology and Cell Science, Institute of Food and Agricultural Sciences, University of Florida, Gainesville, United States of America; 2 Departamento de Genética Molecular, Instituto de Fisiología Celular, Universidad Nacional Autónoma de México, México D.F., México; 3 Department of Microbial Infection and Immunity, The Ohio State University, Columbus, OH, United States of America; University of Kansas Medical Center, UNITED STATES

## Abstract

The two-component signal transduction system BarA-UvrY of *Escherichia coli* and its orthologs globally regulate metabolism, motility, biofilm formation, stress resistance, virulence of pathogens and quorum sensing by activating the transcription of genes for regulatory sRNAs, e.g. CsrB and CsrC in *E*. *coli*. These sRNAs act by sequestering the RNA binding protein CsrA (RsmA) away from lower affinity mRNA targets. In this study, we used ChIP-exo to identify, at single nucleotide resolution, genomic sites for UvrY (SirA) binding in *E*. *coli* and *Salmonella enterica*. The *csrB* and *csrC* genes were the strongest targets of crosslinking, which required UvrY phosphorylation by the BarA sensor kinase. Crosslinking occurred at two sites, an inverted repeat sequence far upstream of the promoter and a site near the -35 sequence. DNAse I footprinting revealed specific binding of UvrY *in vitro* only to the upstream site, indicative of additional binding requirements and/or indirect binding to the downstream site. Additional genes, including *cspA*, encoding the cold-shock RNA-binding protein CspA, showed weaker crosslinking and modest or negligible regulation by UvrY. We conclude that the global effects of UvrY/SirA on gene expression are primarily mediated by activating *csrB* and *csrC* transcription. We also used *in vivo* crosslinking and other experimental approaches to reveal new features of *csrB/csrC* regulation by the DeaD and SrmB RNA helicases, IHF, ppGpp and DksA. Finally, the phylogenetic distribution of BarA-UvrY was analyzed and found to be uniquely characteristic of γ-Proteobacteria and strongly anti-correlated with *fliW*, which encodes a protein that binds to CsrA and antagonizes its activity in *Bacillus subtilis*. We propose that BarA-UvrY and orthologous TCS transcribe sRNA antagonists of CsrA throughout the γ-Proteobacteria, but rarely or never perform this function in other species.

## Introduction

The ability of bacteria to flourish under diverse environmental conditions requires their physiology and metabolism to be regulated by complex transcriptional and posttranscriptional circuitries. The Csr (carbon storage regulator) or Rsm (repressor of stationary phase metabolites) system is a post-transcriptional regulatory system of *E*. *coli* and other γ-proteobacteria that is extensively interconnected with transcriptional regulatory circuits [1–8]. Its centerpiece, CsrA, is a small dimeric RNA binding protein that regulates mRNA translation, turnover and transcription termination [3, 9–11]. CsrA activity in *E*. *coli* and *Salmonella enterica* serovar Typhimurium (hereafter referred to as *Salmonella*) is regulated by CsrB and CsrC sRNAs, which utilize multiple CsrA binding sites to sequester CsrA away from its lower affinity mRNA targets [3, 12–14]. CsrB/C levels are regulated by factors affecting both their synthesis and turnover [15].

CsrB and CsrC transcription is directly regulated by the BarA-UvrY or BarA-SirA two-component signal transduction system (TCS) in *E*. *coli* [13, 16, 17] and *Salmonella* [14, 18, 19], respectively. The BarA protein belongs to a family of membrane-associated tripartite sensor-kinases and UvrY belongs to the FixJ family of response regulators [14, 19–21]. BarA is required for sensing the presence of acetate, formate and other carboxylate compounds by an undetermined mechanism [22–24]. This leads to autophosphorylation at a conserved histidine residue (His^302^), and transphosphorylation of UvrY/SirA through a His^302^→ Asp^718^→ His^861^→ Asp^54^ phosphorelay [20, 22]. Phosphorylated UvrY-P/SirA-P, in turn, binds to its DNA targets and regulates their transcription. For instance, *in vitro* binding studies suggest that the SirA protein (SirA-P) binds to DNA sequences located upstream of target genes for Csr sRNAs and activates their transcription in *Salmonella* [18, 19, 25].

BarA-UvrY and its orthologs in other γ*-*proteobacteria, including GacS/GacA (*Pseudomonas*), VarS/VarA (*Vibrio)*, ExpS/ExpA (*Pectobacterium)* and LetS/LetA (*Legionella pneumophila*) have been reported to regulate virulence, metabolism, biofilm formation, stress resistance, quorum sensing and secretion systems [4, 20, 26–30]. Transcriptomics studies of UvrY and its orthologs have shown effects on the expression of numerous genes [30–32]. An understanding of which of these effects represents direct regulation vs. indirect regulation via effects on the Csr sRNAs is necessary for modeling of the complex genetic circuitry that underpins the systems biology of these species. This question has been examined in only one case, which used ChIP-on-chip analysis to conclude that only the genes for Csr sRNAs (RsmY, RsmZ) in *Pseudomonas aeruginosa* are direct targets for GacA binding [33].

Here, we used ChIP-exo, an advanced procedure for determining genome-wide DNA-protein interactions with single nucleotide resolution [34] to map the UvrY and SirA DNA binding sites across the *E*. *coli* and *Salmonella* genomes. The *csrB* and *csrC* genes were by far the strongest direct targets of binding in both species. These genes exhibited crosslinking at two distinct locations. By comparing the results of *in vivo* crosslinking with *in vitro* DNA binding assays, an 18 NT palindrome or inverted repeat sequence (IR) located far upstream of the promoter was found to serve as a specific binding site for UvrY at *csrB* and *csrC*. Disruption of *barA* eliminated UvrY binding *in vivo*. IHF (integration host factor) was required for optimal UvrY binding to and transcriptional activation of *csrB* but not *csrC*. In addition to the *csrB/C* genes, weaker genomic binding sites of UvrY were identified by ChIP-exo. Several genes associated with the weaker binding sites were tested and found to exhibit negligible or only modest regulation by UvrY, suggesting that diverse effects of this TCS are mediated through Csr circuitry.

In addition to BarA-UvrY, other factors activate *csrB/C* transcription in *E*. *coli*, such as CsrA [13, 16, 35], DksA and ppGpp of the stringent response [2], and the DEAD-box RNA helicases DeaD and SrmB [4]. The stringent response describes a regulatory network of eubacteria that responds to amino acid starvation and other stresses [2]. Activation of this system is characterized by a rapid downshift in synthesis of stable RNAs, such as rRNA and tRNA and stimulation of the expression of genes involved in amino acid biosynthesis and transport, although these processes represent a fraction of its global regulatory role [36]. The effector of this response is the nucleotide secondary messenger guanosine tetraphosphate, ppGpp, also known as “magic spot” [37]. Complexed with RNA polymerase, ppGpp positively or negatively affects transcription [38]. In most cases, regulation by ppGpp requires the RNA-polymerase associated transcription factor DksA [39, 40], which acts together with ppGpp to synergistically regulate expression of a number of global regulators and other genes. The DEAD-box RNA helicases are enzymes that utilize ATP energy to alter RNA structure or RNA-protein interactions [4]. Their roles in the posttranscriptional regulation of bacterial gene expression are presently underappreciated. DeaD regulates *uvrY* translation by altering mRNA structure, while SrmB uses a distinct, but incompletely understood mechanism. We used a combination of *in vivo* and *in vitro* approaches to refine our understanding of how these regulatory factors affect *csrB/C* transcription.

Bioinformatics analyses revealed that BarA-UvrY orthologs are strongly anti-correlated with the *fliW* gene, which encodes a protein that binds to and antagonizes CsrA of *B*. *subtilis* [41], indicating that few if any species use both FliW and BarA-UvrY transcribed sRNAs as CsrA antagonists. These studies advance our understanding of this global regulatory circuitry and highlight the importance of including post-transcriptional regulation along with transcriptional control when modeling bacterial regulatory networks.

## Experimental Procedures

Bacterial strains, plasmids and primers used in this project are listed in S1 Table.

### Bacterial strains and culture conditions

LB medium (1% [w/v] Tryptone; 1% [w/v] NaCl and 0.5% [w/v] yeast extract) was used for culture of bacteria unless stated otherwise. The antibiotics ampicillin (100 μg mL−1), tetracycline (15 μg mL−1), kanamycin (50 μg mL−1), and chloramphenicol (25 μg mL−1) were included in growth media as needed. Bacterial cultures were stored at -80°C in medium containing ~15% glycerol. To revive cultures, LB medium (2 mL) was inoculated from the frozen stock cultures and incubated with shaking (250 rpm) at 37°C overnight. The overnight cultures served as inoculum (1:1000) for LB medium or Kornberg medium (1.1% [wt/vol] K_2_HPO_4_, 0.85% [wt/vol] KH_2_PO_4_, 0.6% [wt/vol] yeast extract containing 0.5% [wt/vol] glucose). Growth was determined by monitoring OD_600_ and/or by assaying total cellular protein.

### Construction of chromosomal deletions and FLAG^®^ fusion proteins

Gene deletions (of *E*. *coli*) and carboxy-terminally 3XFLAG^®^-tagged constructs (of *E*. *coli* and *Salmonella*) were introduced by the Red recombinase method, as described [42, 43]. For gene deletions, primers carrying sequences (40 nt) for the beginning (forward primer) or end (reverse primer) of the open reading frame were designed (S1 Table) and plasmids pKD13 (or pKD3) were used as template DNAs for PCR amplification [43]. For constructing 3XFLAG tag constructs, primers carrying sequences (40 nt) matching the terminus of the targeted gene (forward primer) and the region downstream from it (reverse primer) were designed (S1 Table) and plasmid pSUB11 was used as template DNA for PCR amplification [42, 43]. PCR products were gel purified and used directly for electro-transformation. *E*. *coli* and *Salmonella* strains carrying pKD46 helper plasmid were grown at 30°C in SOB (0.5% [w/v] yeast extract; 2% [w/v] tryptone; 10 mM NaCl, 2.5 mM KCl; 10 mM MgCl_2_; 10 mM MgSO_4_) supplemented with 100 μg/mL ampicillin and 10 mM arabinose to mid-log (OD_600_ of 0.5), collected by centrifugation, washed three times with ice-cold 10% glycerol and resuspended with ice-cold 10% glycerol. The transformation was then performed by electroporation. After 1h recovery at 37°C in SOB medium, bacteria were spread onto LB agar plates supplemented with antibiotics for the selection of Cm^R^ or Kn^R^ recombinants. Correct insertion of the marker and 3XFLAG sequence in the genome was confirmed by PCR amplification and by sequencing of the insertion site using primers listed in (S1 Table). When necessary, the FRT-flanked antibiotic resistance cassette was removed using pCP20 [43].

### Construction and purification of carboxy-terminally His-tagged UvrY protein

His-tagged UvrY (UvrY-His_6_) protein was constructed by PCR amplification of the coding sequence of *uvrY* gene using genomic DNA of *E*. *coli* MG1655 as template DNA and oligonucleotides UvrY-6xhis-F and UvrY-6xhis-R as primers (S1 Table). The amplicon was gel-purified, digested using *Nde* I and *Xho* I restriction enzymes (NEB), and cloned into a similarly digested and dephosphorylated pET24-a (+) vector DNA (NEB) using electrocompetent DH5α for the transformation. The pET-UvrY plasmid was confirmed by gel electrophoresis and sequencing of the inserted DNA using T7-promoter and T7 terminator primers. The pET-UvrY plasmid was moved into BL21 (DE3) *E*. *coli* strain for expression. The *E*. *coli* BL21_DE3/pET-UvrY cells carrying UvrY-His_6_ were grown in 500 mL of LB containing 50 μg/mL kanamycin at 37°C. At OD_600_ of 0.6, expression of the UvrY-His_6_ was induced by the addition of isopropyl β-D-1-thiogalactopyranoside (IPTG) to 1 mM, followed by 2h of shaking incubation at 37°C, 250 rpm.

The purification procedure for UvrY-His_6_ was similar to the method previously described for isolation of DeaD-His_6_ with some modification [4]. Cells were collected by centrifugation (600 x G, 4°C, 5min), suspended in buffer (20 mM Tris-HCl [pH 7.9], 500 mM NaCl, 20 mM imidazole) containing a protease inhibitor cocktail (cOmplete Mini, EDTA-free protease inhibitor cocktail, Roche Diagnostics), and lysed using a French Press. The lysates were centrifuged (20,000 x G, 15 min, 4°C) to remove unbroken cells and cell debris and the supernatant solutions were purified by affinity chromatography on a HisTrap HP column as instructed by the manufacturer (GE Healthcare Life Sciences). Fractions containing UvrY were pooled and dialyzed against UvrY storage buffer (20 mM Tris-HCl (pH 7.9), 150 mM NaCl, 1 mM dithiothreitol and 10% glycerol). UvrY was then aliquoted, frozen using liquid nitrogen, and stored at −80°C. The bicinchoninic acid method was used for assaying protein concentration, as recommended (Pierce Biotechnology).

### UvrY phosphorylation *in vitro*

For *in vitro* phosphorylation, the purified UvrY-His_6_ protein was incubated with 100 mM acetyl-phosphate (Sigma) in a phosphorylation buffer containing 50 mM HEPES pH7.5, 100 mM NaCl and 10mM MgCl_2_. The reaction mixtures were incubated for 60 min at room temperature. To determine relative amount of UvrY-P formed, the reactions were fractionated on Phos-tag^TM^ SDS-PAGE gels [1.0mm Protean 3 (Bio-Rad) minigels] of the following composition: 7.5% separating gel [7.5% (29:1) acrylamide: bis-acrylamide, 357mM Bis-Tris, pH 6.8, 100 μM Zn (NO_3_)_2_, and 50 μM Phos-tag reagent (Waco Pure Chemical Industries)] with a 4% stacking gel [4% (29:1) acrylamide:bis-acrylamide, 357 mM Bis-Tris, pH 6.8]. UvrY-P was resolved by electrophoresis at constant 150 V at 4°C for 70 min using modified MOPS running buffer (100 mM Tris, 100 mM MOPS, 0.5% SDS, and 5 mM NaHSO_3_). Gels were subsequently stained with Coomassie blue and the signals were imaged using a ChemiDoc XRS+ system (Bio-Rad) and quantified using Quantity One image analysis software (Bio-Rad).

### ChIP-exo and deep sequencing

*E*. *coli* and *Salmonella* strains carrying biologically functional UvrY-FLAG [4] or SirA-FLAG proteins (S1 Fig) were grown in Kornberg medium and LB (supplemented with 10 mM glucose), respectively at 37°C, 250 rpm. At mid-exponential phase of growth (OD_600_ of 0.6), formaldehyde (1% final concentration) was added and the cultures were incubated for 20 min at 30°C, 150 rpm. The crosslinking reaction was then quenched by the addition of 5 mL of 1.0 M glycine, pH 8.0 to 10 mL of culture. The samples were kept at room temperature for 5 min with gentle swirling. The cells were harvested by centrifugation (6000 x G, 5 min, 4°C), washed twice with ice-cold 1x PBS, suspended in 500 μL lysis buffer (10 mM Tris-HCl pH 8.8, 50 mM NaCl, 20% [w/v] sucrose, 10 mM EDTA) containing protease inhibitor cocktail (cOmplete Mini, EDTA-free protease inhibitor cocktail, Roche Diagnostics) and 2 mg/mL lysozyme. After 30 min on ice, 500 μl of 2x IP buffer (100 mM Tris-HCl, pH 7.0, 300 mM NaCl, 2% Triton X-100, 2 mM EDTA) was added and the samples were incubated at 37°C for 10 min, followed by 2 min on ice. The samples were then sonicated on ice, (60 pulses total, 5 sec on 5 sec off per pulse, in a total of 6 sets, separated by 2 min on ice, using a sonicator (Fisher Scientific, Sonic Dismembrator Model 500, set at 20% amplitude). Unbroken cells and debris were removed by centrifugation. Sonicated samples (1 mL) were treated with 20 μL ANTI-FLAG® M2 beads (Sigma) by spinning on a tube rotating mixer (Thermo Scientific) at 4°C for 4 hr to immunoprecipitate UvrY-FLAG. The beads were washed three times with 1mL 1X IP buffer and two times with 1mL 1X TE buffer (10 mM Tris-HCl pH 8.0, 1 mM EDTA). The beads were resuspended in 100 μL TE buffer and the formaldehyde crosslinking was reversed by heating at 95°C for 20 min. Afterward, the samples were incubated with 8 μL of 10 mg/mL RNase A for 2 h at 37°C and with 4 μL of 20 mg/mL proteinase K at 55°C for 2h. The resulting DNA was purified using a Qiagen MinElute PCR Purification Kit according to manufacturer’s instructions. As a control, wild type strains expressing no FLAG epitope tagged protein were treated using the same procedures. The specificity of the ChIP assay was confirmed by amplifying promoter DNA of the *csrB*, *lacY* and 16s rRNA (*rrsH*) genes with primers (S1 Table) using the DNA recovered from the ChIP reactions as a template (S2 Fig). *csrB* was used as a positive control for UvrY/SirA binding while *lacY* (*E*. *coli*) and 16S rDNA (*Salmonella*) were used as negative controls. After confirming the specificity of the ChIP reactions, the DNA samples were frozen and shipped to Peconic LLC for the remainder of the ChIP-exo procedure and deep sequencing with Illumina Hi-seq 2000 as described previously [34].

### ChIP-exo data analysis

Sequencing reads were mapped to their respective *E*. *coli* MG1655 (NC_000913.3) and *Salmonella enterica* subsp. *enterica* serovar Typhimurium 14028S (NC_016856.1) genomes with BWA (Burrows-Wheeler Aligner, available from http://bio-bwa.sourceforge.net). Alignments were visualized using the Integrated Genome Viewer (IGV, Broad Institute) [44, 45]. Peaks were identified in the mapped reads with GeneTrack [46]. Low confidence singleton peaks and those without matching peaks on the opposing strand were discarded. Enriched genomic loci were classified into three groups based on enrichment of read counts relative to read counts within 500 bp upstream of the transcription start sites of *lacY* and/or 16s rRNA (*rrsH*), which were determined not to be direct targets of UvrY/SirA (S2 Fig). Maps of enriched regions are shown as bedgraphs, which display in a continuous fashion the abundance of reads identified at particular position in the genome from the sequencing analysis. For motif discovery, DNA sequences from the peak-pair midpoint of each of the enriched genomic loci, with +/- 40nt upstream and downstream extension distance were extracted. Centered in the 81nt-long extracted DNA sequence is the UvrY/SirA crosslinking site. The extracted DNA sequences were uploaded into MEME Suite [47] for motif discovery analysis. The datasets from ChIP-exo analyses were submitted to the NCBI GEO repository under the accession number GSE74810.

### RNA extraction and Northern blotting

Procedures for RNA extraction and Northern blotting were conducted as described previously, with minor modification [4]. Briefly, bacteria were harvested at mid-exponential phase (OD_600_ of 0.6) or as otherwise stated and total cellular RNA was prepared (RNeasy mini kit, Qiagen). RNA was separated by electrophoresis on 5% polyacrylamide gels containing 7 M urea, transferred to a positively charged nylon membrane (Roche Diagnostics) by electro-blotting, and crosslinked to the membrane with UV light. Crosslinked RNA was hybridized to DIG-labeled antisense probe (68°C, overnight) and signal was developed using the DIG Northern Starter kit (Roche Diagnostics). Antisense RNAs were prepared from PCR products using the DIG Northern Starter kit (Roche Diagnostics). Blots were imaged using a ChemiDoc XRS+ system (Bio-Rad) and RNA signals quantified using Quantity One image analysis software (Bio-Rad). The 5S or 16S and 23S rRNAs served as loading controls, and were detected by hybridization or methylene blue staining, respectively.

### Western blotting

Western blotting was conducted as previously described [4]. Briefly, proteins (10 μg) were separated using SDS-PAGE and electroblotted onto 0.2 mm polyvinylidene difluoride membranes.Anti-FLAG® M2 monoclonal antibody (Sigma) and anti-RpoB monoclonal antibody (Neoclone) were used for detection of the FLAG epitope and RpoB. Signal detection used treatment with horseradish peroxidase-linked secondary antibodies followed by SuperSignal® West Femto Chemiluminescent Substrate (Thermo Scientific). Blots were imaged using the ChemiDoc XRS+ system (Bio-Rad) and the signals were quantified using Quantity One image analysis software (Bio-Rad).

### Analysis of UvrY-FLAG protein stability

Bacterial cells were grown to mid-exponential phase of growth, at which point protein synthesis was stopped by the addition of tetracycline (200 μg/mL) and chloramphenicol (100 μg/mL). Cells were collected at several times following the addition of the antibiotics, harvested by centrifugation and were immediately mixed with Laemmli sample buffer and lysed by sonication and boiling. Samples (10 μg protein) were subjected to SDS- PAGE and were analyzed by Western blotting as described above.

### β-Galactosidase assays

The assays for specificβ-Galactosidase activity were conducted as described previously [2]. Values represent the averages from two independent experiments. Error bars represent standard errors of the means.

### Analysis of UvrY phosphorylation *in vivo*

Studies to determine the extent of phosphorylation of the UvrY-FLAG protein *in vivo* were conducted as previously described [4]. Briefly, proteins from cell lysates were fractionated on Phos-tag^TM^ SDS-PAGE gels, which resolve UvrY from UvrY-P, and the FLAG epitope of the UvrY-FLAG protein was detected by Western blotting.

### Quantitative RT-PCR

Quantitative Real Time-PCR (q-RT-PCR) was conducted in an iCycler™ thermocycler (Bio-Rad) using the iScript™ One-Step RT-PCR Kit with SYBR® Green (Bio- Rad) as described previously, with minor modifications [4]. Reactions (15 μl) contained immunoprecipitated DNA (50 ng/μl), primers (0.5 μM each; S1 Table) and SYBR® Green RT-PCR Reaction Mix. PCR cycle parameters were as follows: 40 cycles of PCR at 95°C denaturation for 10 s, 60°C of annealing, extension, and detection for 30 s. The specificity of the PCR product was determined by melting curve analysis with reference to the calculated Tm. For melting curve analysis, the temperature was increased from 60°C to 95°C at a rate of 0.5°C/10 s. PCR product concentration was determined using a standard curve prepared with iCycler iQ optical system software version 3.1 (Bio-Rad), according to the manufacturer’s instructions.

### Analysis of UvrY binding to *csrB* DNA *in vivo* by ChIP-PCR

UvrY binding to *csrB* genomic DNA *in vivo* was analyzed by ChIP-quantitative PCR (ChIP-PCR). The first steps of the ChIP-PCR assay were conducted as described for the ChIP-exo assay. After releasing the formaldehyde crosslinks, the DNA was extracted and purified using Qiagen MinElute Purification Kit. The isolated *csrB* DNA was assayed using quantitative real time-PCR (q-RT-PCR) with primers listed in (S1 Table). The *lacY* gene served as a negative control for this reaction using primers also listed in (S1 Table).

### Electrophoretic gel mobility shift assay (EMSA) for DNA binding

For DNA electrophoretic gel mobility shift assays, the regions from -246 to +49 and -247 to +56, with respect to the transcriptional start sites of *csrB* and *csrC*, respectively, were amplified by PCR and end-labeled with [γ-^32^P] ATP using T4 polynucleotide kinase. Binding reactions (10 μl) contained 0.5 nM of end labeled DNA, 20 mM Tris HCl (pH 7.5), 10% (v/v) Glycerol, 50 mM KCl, 3 mM MgCl_2_, 1 mM dithiothreitol, 100 μg/mL BSA and phosphorylated or non-phosphorylated UvrY-His_6_ protein. Reactions were incubated for 30 min at 37°C, then 1μl xylene cyanol was added and samples were separated by electrophoresis on 7% non-denaturing polyacrylamide gels with 0.5X TBE as the running buffer. After electrophoresis, the gels were vacuum dried, the radioactive signals captured by phosphorimage analysis (PMI^TM^, Bio-Rad) and analyzed using Quantity One software.

### DNase I footprinting

Double stranded DNA for footprinting of *csrB* (466 bp long, extending from 420 bp upstream to 46 bp downstream of the TSS) or *csrC* (419 bp long, extending from 319 bp upstream to 100 bp downstream of the TSS) was prepared by PCR using strand-specific [^32^P] 5’-end-primers (S1 Table). The 5’-end-labeled PCR products were gel-purified and the DNA (0.5 nM) was used for binding reactions (10 μL) containing 20 mM Tris HCl (pH 7.5), 10% (v/v) glycerol, 50 mM KCl, 3 mM MgCl_2_, 1mM dithiothreitol and 100 μg/mL bovine serum albumen along with phosphorylated or non-phosphorylated UvrY-His_6_ protein. The binding reactions were incubated at 37°C degrees for 30 min followed by treatment with 0.025 U DNase I (Roche) per reaction, for 1 min at 37°C. The DNase I was inactivated by heating at 75°C degrees for 10 min. Reactions were then mixed with formamide loading buffer (10 mL formamide, 10 mg xylene cyanol FF and 10 mg bromophenol blue), denatured at 95°C for 5 min, and separated by electrophoresis on a 6% denaturing polyacrylamide gel. The gel was vacuum dried, radioactive signals captured by phosphor imaging (PMI^TM^, Bio-Rad) and analyzed with Quantity One software.

### Construction of plasmid-borne *csrB-lacZ* and *csrC-lacZ* fusions for S-30 transcription-translation

The *csrB-lacZ* and *csrC-lacZ* carrying plasmids, pLFXcsrB-lacZ and pLFXcsrC-lacZ, were constructed by first amplifying the 502 nt (-500 to +2 with respect to *csrB* transcriptional start site) and 304 nt (-301 to +3 with respect to *csrC* transcriptional start site) genomic regions using the primer pairs csrB lacZ Fwd / csrB lacZ Rev and csrC lacZ Fwd / csrC lacZ Rev (S1 Table). The PCR products were gel purified, digested with *Pst*I and *Kpn*I, ligated to *Pst*I- and *Kpn*I-digested and dephosphorylated plasmid pLFX, and electroporated into DH5αλpir cells. DNA sequencing was used to confirm that the cloned regions did not contain any mutations.

### *In vitro* coupled transcription-translation

*In vitro* transcription-translational assays used pLFXcsrB-lacZ and pLFXcsrC-lacZ supercoiled plasmids and were performed with S-30 extracts prepared from the UvrY deficient strain, CF7789 *uvrY*::*cam*, as described previously [16], except that reactions (32 μl) contained 0.5 U *E*. *coli* RNA polymerase holoenzyme and 3 μl of ^35^S-methionine (1175 Ci/mmol). The UvrY-P (2.3 μM), ppGpp (250 μM) and DksA (2 μM) were included, as indicated, in the reactions. IHF (1μM) was added to all of the reactions that contained pLFXcsrB-lacZ plasmid. DksA protein was a generous gift of Prof. Richard Gourse, University of Wisconsin, Madison. IHF protein was a generous gift from Prof. Anca Segall, San Diego State University. Incorporation of ^35^S-methionine into protein products was determined after SDS PAGE separation by using phosphorimaging. Signal intensity was determined using Quantity One software.

### Phylogenetic analysis of BarA, UvrY, CsrA, and FliW distribution

We identified CsrA, BarA, UvrY and FliW orthologs from all fully-sequenced bacterial genomes in the NCBI genomes database (http://www.ncbi.nlm.nih.gov/genome/), using the NCBI Prokaryotic Genome Annotation Pipeline v2.0 to assign orthology [48]. This pipeline combines a sequence similarity-based approach with the comparison of the predicted gene products to the nonredundant protein database, Entrez Protein Clusters, the Conserved Domain Database (CDD) [48].

In order to filter out false positives and false negatives from the NCBI orthologs data set, we aligned a set of representative sequences against the genomes with predicted orthologs using tblastn [49]. The alignment results were filtered by sequence similarity and alignment coverage: BarA was filtered by 50% similarity and 50% coverage, UvrY was filtered by 75% similarity and 80% coverage, CsrA was filtered by 60% similarity and 50% coverage, and FliW was filtered by 50% similarity and 50% coverage. All the similarity and coverage thresholds were established by the minimum similarity and coverage observed between annotated sequences from NCBI nucleotide database and the following set of representatives used as query sequences: BarA from *Escherichia coli* (NCBI protein identification number PI: 190908466), UvrY from *E*. *coli* (PI: 190906390), CsrA from *E*. *coli* and *Bacillus subtilis* (PI: 257755450 and 459391362), and FliW from *Bacillus amyloliquefaciens* (PI: 307608134). After filtering the results by alignment similarity and coverage, we performed a manual refinement of the results by verifying the pairwise alignments, sequence annotations, and bibliographic information.

A reference phylogeny of fully-sequenced genomes encoding CsrA and at least one of BarA, UvrY or FliW was extracted from the NCBI taxonomy database [50] using NCBI-taxcollector [51] and PhyloT v2015.1 (http://phylot.biobyte.de), and gene presence/absence data were visualized using iTOL v3.0 [52]. Correlations among presence/absence of CsrA, BarA, UvrY and FliW were calculated using Pearson’s product moment correlation, and significance was assessed using Fisher’s Z transform (implemented in the R stats package, v3.2.0). For calculating the correlations, we clustered the results at species and genus level in order to minimize the number of false negatives and false positives. For clustering the results at species level, we established the presence/absence as the value that appears most often at strain level (statistical mode). For clustering the results at genus level, we established the presence/absence as the mean calculated at species level.

In order to determine if any strong biases in the methodology used to identify orthologous genes could affect our results we used two additional and widely-employed methods to identify alternative ortholog sets: KEGG orthology database (KO) [53], and UniProt reference clusters of orthologs (UniRef) [54]. KEGG database defines the orthologs by comparing experimental data with KEGG pathway maps, BRITE functional hierarchies and KEGG modules. UniProt defines the orthologs by clustering protein sequences by 50% identity. We compared the correlations calculated for these alternative approaches to the results obtained from the NCBI Prokaryotic Genome Annotation Pipeline, at genus and species level.

## Results and Discussion

### Determination of genome-wide UvrY/SirA-binding loci using ChIP-exo

**Fig 1:**
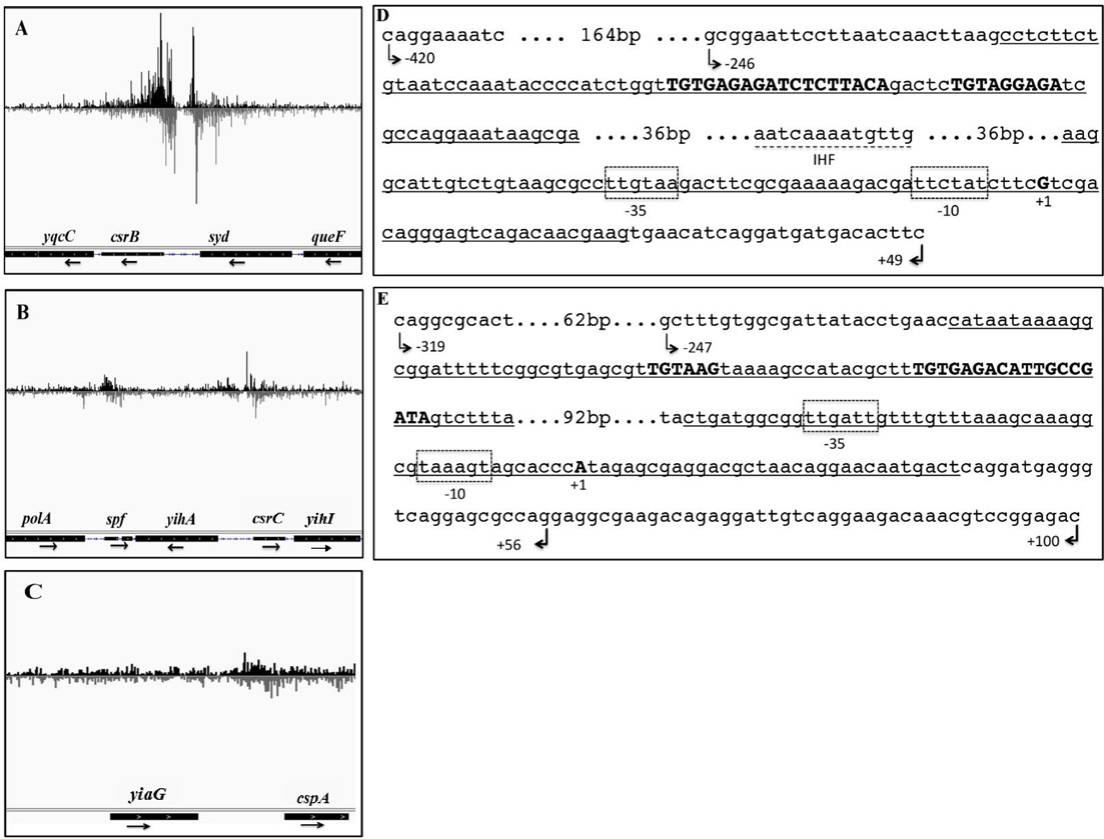
Genomic binding sites for UvrY identified by ChIP-exo. Bedgraphs depict genomic loci enriched by co-immunoprecipitation with cross-linked UvrY-FLAG, proximal to the *csrB* (A), *csrC* (B), *spf* (B) and *cspA* (C) genes. Two UvrY crosslinking sites were discovered in the promoter regions of *csrB* (panel A, underlined in panel D) and *csrC* (panel B, underlined in panel E). One site is within the region extending from -222 to -142 in *csrB* or from -223 to -143 in *csrC*. The other crosslinking site lies close to the promoter, extending from -56 to +25 in *csrB* and from -49 to +32 in *csrC* promoter regions. A 9 bp inverted repeat DNA sequence within the upstream crosslinking regions is bolded and capitalized. A putative IHF binding site (broken underline) is located between the two UvrY crosslinking sites in the promoter region of *csrB* (D), but was not apparent in *csrC*. The DNA used for DNase I footprinting included 466 bp (-420 to +46) for *csrB* and 419 bp (-319 to +100) for *csrC*. DNA fragments used for electrophoretic mobility shift assay included (-246 to +49) for *csrB* and (-247 to +56) for *csrC*. The images were constructed using the Integrated Genome Viewer (IGV, Broad Institute) (44, 45).

**Fig 2:**
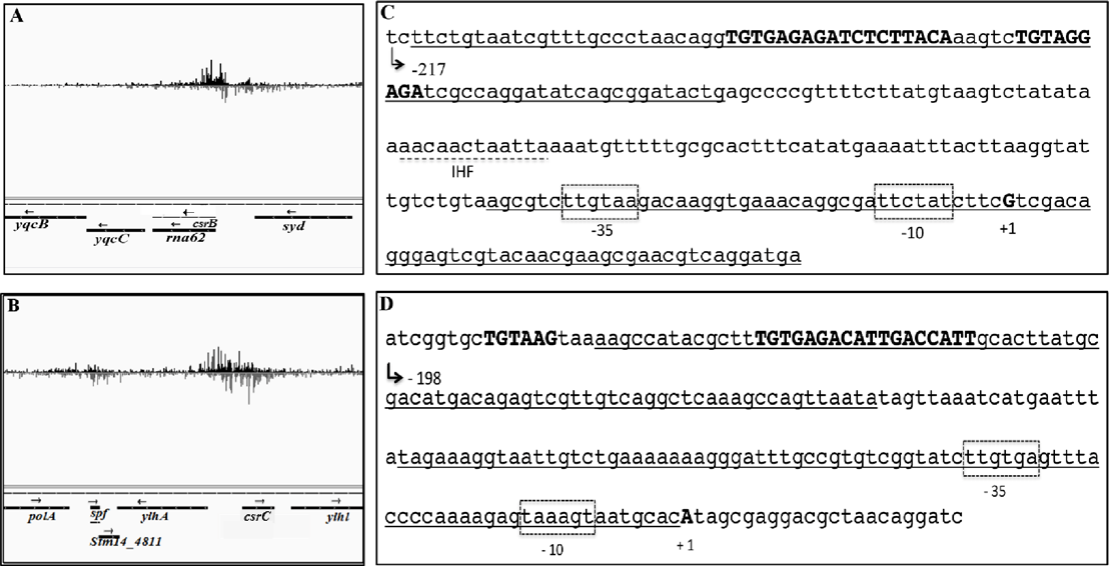
Genomic binding sites for SirA discovered by ChIP-exo. Bedgraphs show enriched genomic loci, proximal to the regulatory regions of *csrB* (A), *csrC* (B) and *spf* (B). Two putative SirA binding sites are shown for the promoter regions of *csrB* (*rna62*) (panel A, underlined in panel C) and *csrC* (panel B, underlined in panel D). One site is located in the region from -215 to -135 in *csrB* and from -181 to -101 *csrC* promoter regions, respectively. The other putative binding site is close to the promoter, within the -41 to +40 (*csrB*) and -81 to -1 in *csrC* promoter regions, respectively. A 9 bp-long inverted repeat DNA sequence in the upstream sites is shown in bold and capitalized. A putative IHF binding site is marked between the two putative UvrY binding sites in the promoter region of *csrB* (C, broken underline), but was not apparent in *csrC*. The images were constructed using the Integrated Genome Viewer (IGV, Broad Institute) (44, 45).

To probe for UvrY/SirA DNA binding sites in *E*. *coli* and *Salmonella*, we used ChIP-exo, a comprehensive genomic DNA-protein interaction assay with single nucleotide resolution and high specificity [34]. This assay uses nuclease trimming reactions to remove nonspecific DNA from the immunoprecipitated complexes and to identify the crosslinking sites much more precisely than ChIP-seq [34]. The experiments were performed with mid-exponential phase cultures (OD_600_ of 0.6) of strains carrying FLAG-tagged UvrY or SirA proteins, respectively, expressed from the native genomic loci. Altogether, 44 million sequencing reads for UvrY (*E*. *coli*) and 31 million for SirA (*Salmonella*) were mapped to their respective genomes. From these analyses, two highly enriched genomic loci were identified at the *csrB* and *csrC* genes of both *E*. *coli* (Fig 1A and 1B *)* and *Salmonella* (Fig 2A and 2B *)*. These results indicated that the *csrB* and *csrC* genes represent the strongest targets of UvrY/SirA binding in these species.

In addition to the *csrB* and *csrC* genes, weakly enriched genomic loci were identified proximal to the promoter regions of 286 genes in *E*. *coli* and 301 genes in *Salmonella* (S2 Table). These enriched genomic loci were arbitrarily classified into three groups based on enrichment of their occupancy peaks over background (S2 Table). Accordingly, genomic loci enriched 5-fold or greater than the *lacY* and/or 16s rRNA (*rrsH*) genes were included in group one. Besides *csrB* and *csrC*, the promoter regions of *fhuF* and *spf* from *E*. *coli* and *spf* from *Salmonella* fell into group one. Genomic loci that were enriched 2- to 5-fold comprised group two, which included the promoter regions of 9 genes in *E*. *coli* and 8 genes in *Salmonella*. The rest of the genomic loci, which include regions proximal to the promoter regions of 275 genes in *E*. *coli* and 292 genes in *Salmonella*, have only1.5- to 2.0 occupancy read fold over that of *lacY* or 16s rDNA (*rrsH*) and were grouped into group three.

### UvrY/SirA consensus DNA binding motif

Further inspection of the ChIP-exo results showed enriched crosslinking in the upstream locations of *csrB* and *csrC*, with sequences extending from -222 to -142 from the start of transcription (*csrB*) or -223 to -143 (*csrC*) in *E*. *coli* (Fig 1D and 1E) and from -215 to -135 (*csrB*) and -181 to -101 (*csrC*) in *Salmonella* (Fig 2C and 2D). Distinct sites of crosslinking in these genes were also observed at downstream sequences that extended from -56 to +25 (*csrB*) and from -49 to +32 (*csrC*) in *E*. *coli* (Fig 1D and 1E) and from -41 to +40 (*csrB*) and from -81 to -1 (*csrC*) in *Salmonella* (Fig 2C and 2D *)*. In the center of each of the upstream putative binding sites is an 18 nt nearly perfect palindrome or inverted repeat sequence (IR). In *csrB*, the sequence, TGTGAGAGATCTCTTACA, is centered at -183/-182 in *E*. *coli* (Fig 1D) and -182/-181 in *Salmonella* (Fig 2C). Moreover, a partially conserved additional sequence (TGTAGGAGA) located 5 bp downstream of the IR, is seen in the *csrB* promoter of *E*. *coli* (Fig 1D *)* and *Salmonella* (Fig 2C *)*. The IR of *csrC* shows weaker symmetry in both *E*. *coli* (TGTGAGACATTGCCGATA) (Fig 1E) and *Salmonella* (TGTGAGACATTGACCATT) (Fig 2D). Moreover, a partially conserved sequence (TGTAAG) representing half of the IR, located 16 bp upstream of the IR, is also seen in the *csrC* promoter of *E*. *coli* (Fig 1E) and *Salmonella* (Fig 2D). In contrast, the IR is not preserved in the downstream crosslinking sites of the *csrB* or *csrC* genes.

To search for the conserved IR at the weaker targets of UvrY/SirA (S2 Table), DNA sequences from these enriched promoter regions were analyzed using MEME Suite software [47]. These analyses showed that the IR sequence is not conserved in any of the weaker putative binding sites.

### UvrY requires phosphorylation for DNA binding

In the BarA-UvrY TCS, BarA is the histidine kinase, which upon signal detection, autophosphorylates and transfers the phosphoryl group to a conserved aspartate residue of UvrY [20]. The phosphorylated UvrY is then thought to bind specific DNA sequences in the promoters of its target genes and regulate their transcription [18, 20, 22]. MBP-SirA was previously found to bind similarly to *csrB* DNA *in vitro* regardless of whether it had been phosphorylated or not, starting at a concentration of 1.5 μM [25], suggesting that MBP-SirA phosphorylation might not be required for DNA binding *in vitro*. This observation was consistent with another report, showing that phosphorylation only increased the DNA binding affinity of SirA-His_6_ by approximately two-fold *in vitro* [19].

In contrast to results from *in vitro* DNA binding experiments, deletion of the gene for BarA sensor kinase caused more than a 10-fold decrease in the level of CsrB RNA [16, 22, 35]. Similarly, in *Salmonella*, substitution of alanine for the predicted phosphorylated histine residue of SirA, Asp54, caused the loss of CsrB expression [25]. Thus, it appears that phosphorylation is critical for UvrY activity *in vivo*. Whether UvrY requires phosphorylation for efficient DNA binding *in vivo* or for later steps in transcription initiation was not clear.

**Fig 3:**
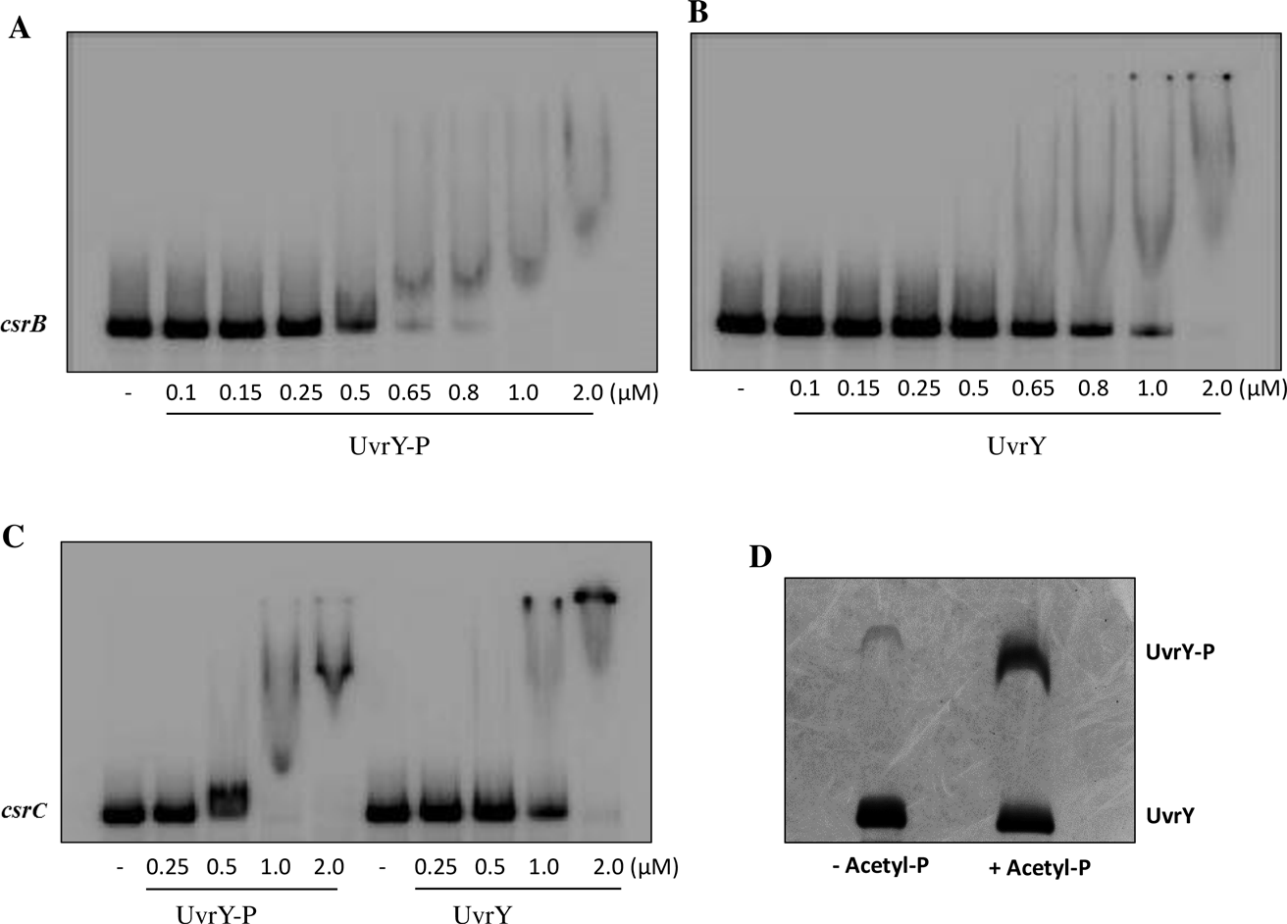
Electrophoretic gel mobility shift assay showing UvrY binding to *csrB* and *csrC* DNA. Binding of phosphorylated (UvrY-P) and non-phosphorylated (UvrY) UvrY-His_6_ to *csrB* DNA (A and B) and *csrC* DNA (C) was tested as shown. The *csrB* and *csrC* DNA probes (0.5 nM) used for this experiment (depicted in Fig 1D and 1E, respectively) were incubated with increasing concentrations of *in vitro* phosphorylated or non-phosphorylated UvrY-His6 protein for 30 min at room temperature. The DNA-protein complexes were resolved by electrophoresis on a non-denaturing 7% polyacrylamide gel. The phosphorylation state of the UvrY-His_6_ protein used in these experiments was determined by Phos-tag SDS PAGE gel analysis (D).

To address this issue, we used both *in vitro* and *in vivo* assays to investigate the role of UvrY phosphorylation in DNA binding. We performed electrophoretic mobility shift assay (EMSA) using phosphorylated (UvrY-P) and non-phosphorylated forms of the recombinant protein, UvrY-His_6_, which was determined to be functional *in vivo* (S1 Fig). DNA fragments that encompass the ChIP-exo-derived putative UvrY binding sites in the promoter regions of *csrB* (Fig 1D) and *csrC* (Fig 1E) were used as probes. Unlabeled *csrB* and *rrlE* DNA fragments were used as competitive and non-competitive DNA probes, respectively. Similar to the previous observations [19, 25], our results showed that *in vitro* phosphorylation led to a modest (~2-fold) increase in DNA binding affinity of UvrY to *csrB* (Fig 3A and 3B) and *csrC* DNA (Fig 3C), as compared to the non-phosphorylated UvrY. However, we also observed that the UvrY protein preparation contained around 7% of UvrY-P prior to the *in vitro* phosphorylation reaction (Fig 3D). It is possible that this contaminating UvrY-P was entirely responsible for binding to *csrB* and *csrC* DNA *in vitro* (Fig 3B).

**Fig 4:**
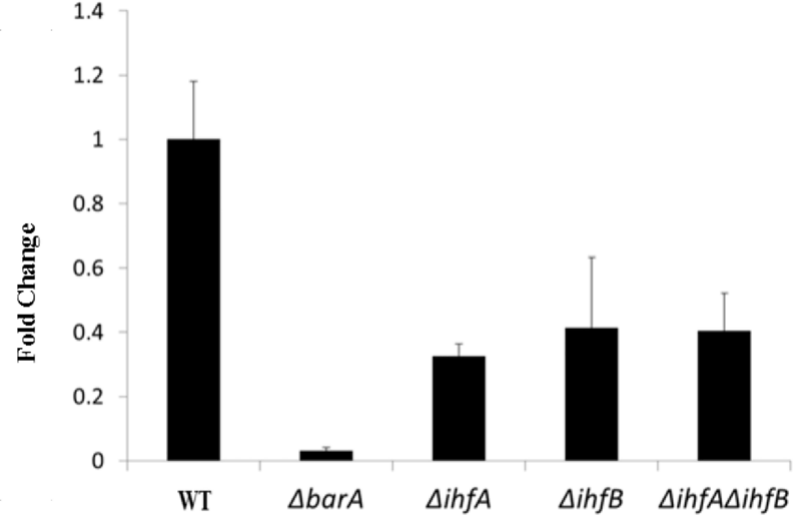
Effects of BarA and IHF on *in vivo* binding of UvrY to *csrB*. The effects of BarA, IhfA and IhfB on *in vivo* binding of UvrY to *csrB* promoter were determined by ChIP-quantitative PCR (ChIP-PCR) in a WT strain (MG1655 expressing UvrY-FLAG) and isogenic ∆*barA*, ∆*ihfA*, ∆*ihfB* and ∆*ihfA*∆*ihfB* strains, as described in the Experimental Procedures. The *lacY* gene served as a negative control for this reaction. Data depict the results of three independent experiments. Error bars represent the standard errors of the means.

To determine whether UvrY requires phosphorylation for *in vivo* DNA binding, we tested UvrY-FLAG binding to *csrB in vivo* in a strain that lacked BarA (∆*barA*) and its isogenic *barA* wild-type strain. For this analysis, we first determined the phosphorylation state of UvrY-FLAG protein in the presence and absence of BarA. The results indicated that in the presence of BarA, approximately 7% of the UvrY protein was phosphorylated (S3 Fig), similar to a previous determination [4]. In the absence of BarA, no detectable phosphorylation of UvrY-FLAG protein was observed in LB medium at mid-exponential phase of growth (S3 Fig). We next used ChIP-PCR to test for *in vivo* binding of UvrY to *csrB* DNA under this growth condition. In this experiment, an approximate 35-fold reduction in DNA binding was observed in the strain that lacked BarA (∆*barA*) relative to the isogenic *barA* wild-type strain (Fig 4). These results show for the first time that UvrY requires phosphorylation for effective DNA binding *in vivo*.

### UvrY specifically binds to an 18 nt-long IR DNA sequence

**Fig 5:**
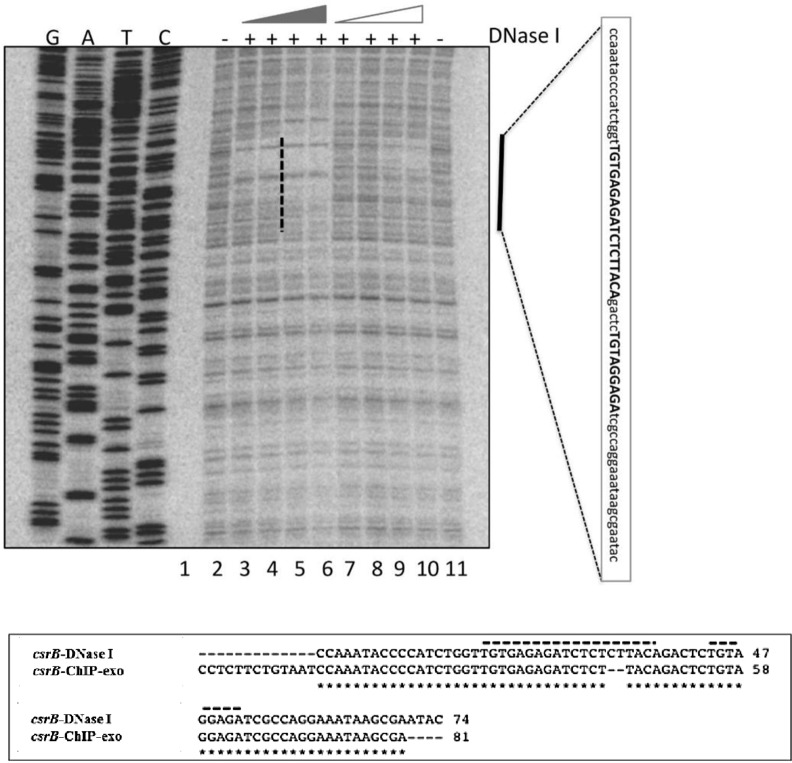
DNase I footprinting of *E*. *coli csrB* DNA using phosphorylated and non-phosphorylated UvrY. A ^32^P-end labeled DNA probe that included both the upstream and downstream putative UvrY binding sites was used for these experiments (shown in Fig 1D). Reactions in all lanes except 1 contained DNase I (0.025U/12.5ul reaction). Reactions in lanes 3–6 and lanes 7–10 contained 0.25, 0.35, 0.5, 0.7 μM of phosphorylated or non-phosphorylated UvrY-His_6_, respectively. Lane 2 reaction contained no UvrY protein. A vertical black bar indicates a protected region, and the sequence corresponding to the protected region is shown in a vertical rectangular box. An alignment of sequences corresponding to the protected regions from DNase I and the ChIP-exo results is shown in the horizontal rectangular box. The 18nt-long palindromic sequence and the partially conserved palindromic sequences are marked with broken black lines.

**Fig 6:**
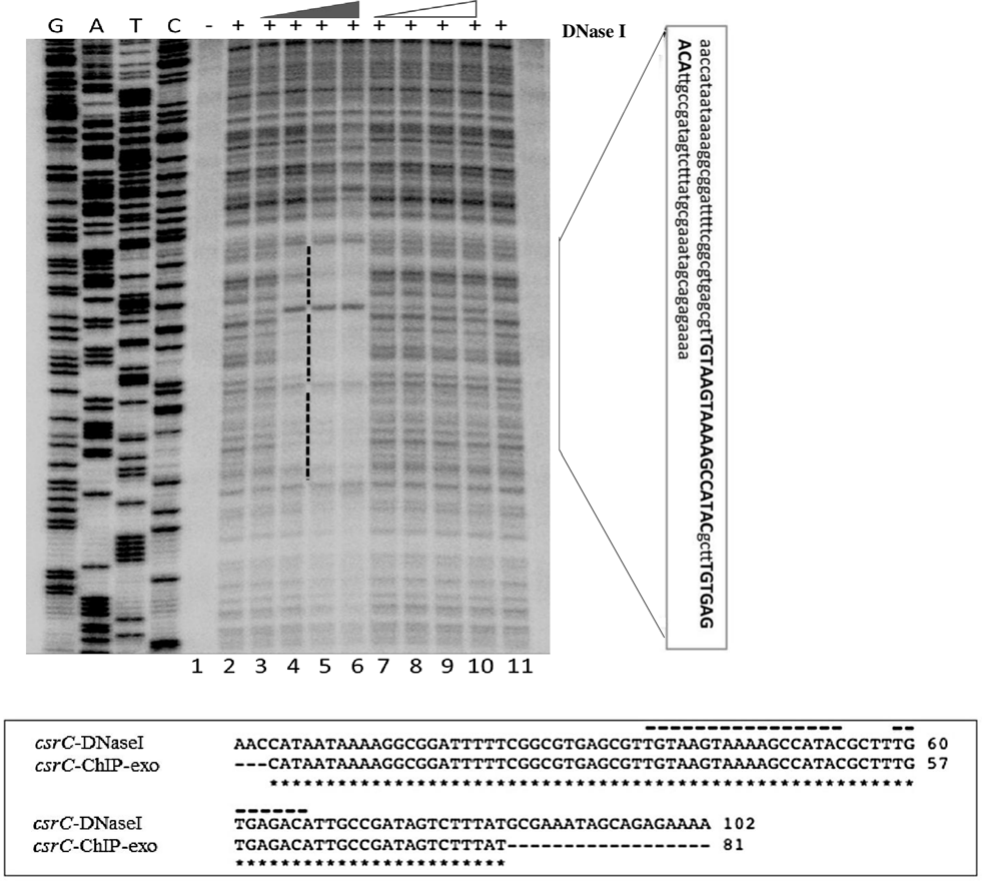
DNase I footprinting of *E*. *coli csrC* DNA by phosphorylated and non-phosphorylated UvrY. A ^32^P-end labeled DNA probe that included both the upstream and downstream putative UvrY binding sites was used for these experiments (Fig 1E). Reactions in all lanes except lanes 1 contained DNase I (0.025U/12.5ul reaction). Reactions in lanes 3–6 and lanes 7–10 contained 0.25, 0.35, 0.5, 0.7 μM of phosphorylated or non-phosphorylated UvrY-His_6_, respectively. Lane 2 reaction contained no UvrY. A vertical black bar indicates a protected region, and the sequence corresponding to the protected region is shown in a vertical rectangular box. An alignment of sequences corresponding to the protected regions from DNase I and ChIP-exo results is shown in the horizontal rectangular box. The 18nt-long palindromic sequence and the partially conserved palindromic sequences are marked with broken black lines.

In order to more precisely define the UvrY DNA binding sites, we performed *in vitro* DNase I footprinting experiments on *csrB* and *csrC* DNA using phosphorylated and non-phosphorylated UvrY-His_6_ protein. The DNA probes for this experiment encompassed both the upstream and downstream putative binding sites of these genes (Fig 1D and 1E). The results of these experiments showed protection of only the upstream binding sites containing the IR sequences of both *csrB* (Fig 5) and *csrC* (Fig 6 and S4 Fig). The putative downstream binding sites that were observed *in vivo* (Fig 1A and 1B & 1D and 1E) were not protected *in vitro*. In addition, only the phosphorylated UvrY-His6 was observed to protect the IR, which further indicates that UvrY requires phosphorylation for tight, specific binding. Together, these results suggest that UvrY-P binds specifically and directly to the IR sequence of these genes. However, *in vivo* binding of UvrY-P to the downstream sequences of these genes either requires conditions or factor(s) that were absent from the footprinting reactions or perhaps more likely, UvrY-P becomes cross-linked to DNA indirectly through interactions with DNA binding proteins such as RNA polymerase, which must bind to the downstream regions of these genes in order to initiate transcription.

### UvrY requires IHF for optimal binding to and expression of *csrB* but not *csrC*

The nucleoid-associated protein Integration Host Factor (IHF) is a heterodimeric protein composed of two homologous subunits, IHFα (IhfA) and IHFβ (IhfB), which facilitates the transcription of many genes by bending the DNA [55–57]. In the Csr/Rsm system, IHF was shown to directly bind to the promoter of the *csrB* gene of *Salmonella*) [18] and the *rsmZ* promoter of *Pseudomonas fluorescens* [58]. In *Salmonella*, deletion of *ihfA* was also shown to decrease *csrB* expression [18]. However, whether the observed effect of IHF in sRNA expression in those bacterial species is UvrY-mediated and if so, whether IHF is required for UvrY to bind to DNA or for later transcription initiation steps is not clear.

**Fig 7:**
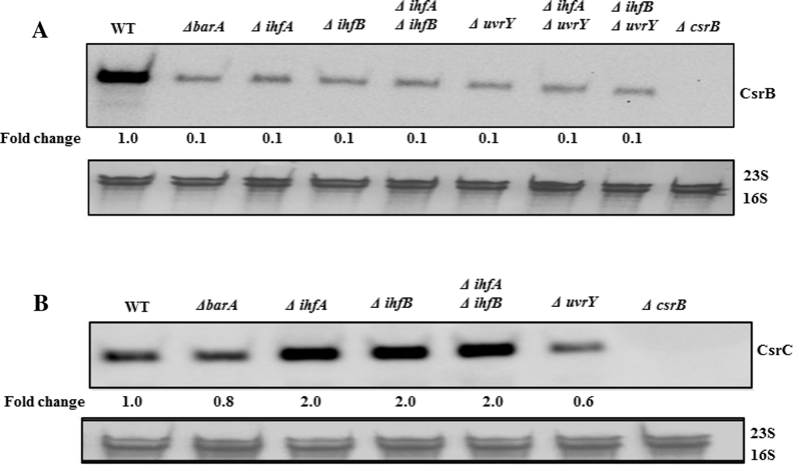
Effects of UvrY, BarA and IHF on CsrB/C sRNAs levels. Northern blots showing effect of several gene deletions on the levels of *E*. *coli* CsrB (A) and CsrC (B). Cultures were grown in LB to mid-exponential growth phase (OD_600_ of 0.6). The 16S/23S rRNA loading controls are also shown.

In this study, we identified a putative IHF binding site in the promoter region of *csrB* of *E*. *coli* (Fig 1D) and *Salmonella* (Fig 2C), in agreement with an earlier report [18], located between the upstream and downstream *in vivo* crosslinking sites for UvrY-P, but we did not identify a similar site within the *csrC* genes. To determine whether IHF affects expression of *csrB* in *E*. *coli*, we measured the levels of CsrB in the presence and absence of IHF (∆*ihfA* and/or ∆*ihfB*). Our results revealed a ~10-fold reduction in CsrB RNA levels in the absence of IhfA, IhfB, or both, compared to the isogenic wild type strain (Fig 7A). We also performed epistasis experiments in which we measured the effect of IHF (∆*ihfA* and/or ∆*ihfB*) on the expression of *csrB* in a strain lacking *uvrY* (∆*uvrY)*. Our results showed that neither IhfA nor IhfB affected the levels of CsrB in the *uvrY* mutant strain (Fig 7A), suggesting that UvrY mediates the effect of IHF on the expression of *csrB*. Analysis of *csrB* DNA binding by UvrY-FLAG using *in vivo* ChIP-PCR analyses showed that binding was reduced by approximately 2.5-fold in the absence of either IhfA or IhfB (Fig 4), suggesting that IHF is required for optimal binding of UvrY-P to *csrB* DNA *in vivo*. However, this modest effect of IHF on UvrY DNA binding does not appear to account for its 10 to 12-fold effect on *csrB* expression. Therefore, IHF also appears to affect later steps in *csrB* transcription (Fig 7A). Western blotting experiments demonstrated that IHF did not affect the phosphorylation (S3 Fig) or expression of UvrY-FLAG (S5 Fig). Hence, our results reveal that IHF is required for optimum binding of UvrY-P to *csrB* DNA and activation of *csrB* transcription.

IHF facilitates the transcription of many genes by bringing relatively distant DNA sites (~200 bp) closer together in space by bending the DNA (~140°) [59, 60]. Therefore, we propose that UvrY-P bound at the upstream IR site (centered at -183/-182 in *csrB* of *E*. *coli* and at -182/-181 in the *csrB* of *Salmonella*) is brought into the proximity of the *csrB* promoter-RNA polymerase complex by IHF-mediated bending of the DNA. Such DNA bending may lead to UvrY-P binding to the downstream DNA crosslinking site directly or indirectly through interactions with RNA polymerase or perhaps with other unknown DNA binding factor(s). This type of transcriptional activation, known as repositioning, is observed in promoters where the primary activator is unable to make a productive contact with RNA polymerase without being repositioned by a secondary activator [60, 61]. Such a mechanism appears to be required for the activation of the *E*. *coli narG* promoter by NarL, another FixJ family response regulator, which binds at –190, while IHF binds at -125 and Fnr binds at – 41 of *narG* [59, 60].

In contrast to its effect on *csrB*, IHF was not needed for expression of *csrC* (Fig 7B). In fact, in the single and double IHF deletion strains, CsrC RNA levels were 2-fold higher than in the isogenic wild type strain (Fig 7B). This may be because in the ∆*ihfA* ∆*ihfB* strains, the lower levels of CsrB RNA (Fig 7A) lead to an increase in the concentration of free CsrA in the cell, which in turn leads to increased levels of CsrC via a negative feedback loop in the Csr system [13].

### UvrY effects on the expression of other putative *in vivo* targets

**Fig 8:**
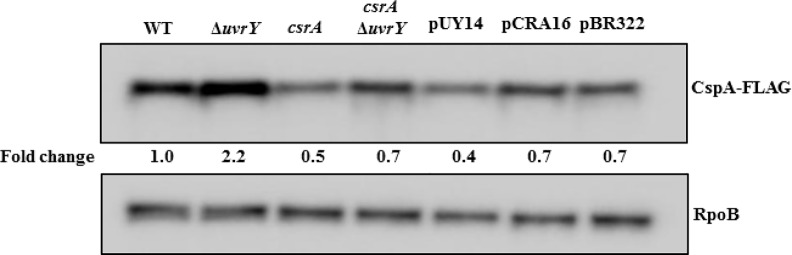
Effects of genes encoding UvrY and Csr factors on CspA protein levels. Western blot showing effects of *uvrY* deletion and *csrA*::*kan* disruption on the levels of CspA-FLAG protein in an MG1655 derivative expressing CspA-FLAG (WT) from the *cspA* genomic locus. Effect of *csrA*::*kan* Δ*uvrY* complemented with UvrY (pUY14), CsrA (pCRA16) or control (pBR322) expression plasmids is also shown. Cultures were grown at 37°C in LB to mid-exponential growth phase (OD_600_ of 0.6). The RpoB protein served as a loading control. This experiment was repeated at least three times with reproducible results.

To examine the regulatory effects of UvrY on the expression of the potential new target genes discovered by ChIP-exo (S2 Table), we tested several gene products and transcripts from groups one, two and three by Western and Northern blotting. Our results showed little or no regulatory effects of UvrY on the expression of the genes that were tested (S6A and S6C Fig), with the exception of *cspA*. UvrY showed a relatively modest, but reproducible negative effect on the expression of the cold-shock protein CspA (Fig 8).

A 2-fold increase in the levels of CspA-FLAG was observed in a Δ*uvrY* strain compared to the isogenic wild type strain (Fig 8). Furthermore, disruption of *csrA* caused a modest decrease in CspA-FLAG levels, suggesting that CsrA may activate *cspA* expression. To determine whether UvrY regulates *cspA* indirectly through its effects on CsrB/C, and therefore CsrA activity, we conducted an epistasis analysis (Fig 8). A strain that was disrupted in both *uvrY* and *csrA* was transformed with plasmids expressing *uvrY* or *csrA* from the plasmid cloning vector pBR322, and CspA-FLAG protein levels were determined in the resulting strains. We observed that CspA-FLAG levels were essentially identical in strains containing pBR322 and the *csrA*-expression plasmid pCRA16. In contrast, the *uvrY*-expression plasmid (pUY14) led to modest decrease in CspA-FLAG levels in this strain. These results are consistent with a model in which UvrY inhibits *cspA* expression by binding to the *cspA* promoter, while the effect of CsrA may be mediated indirectly. Also consistent with this model, *in silico* analyses did not reveal typical CsrA binding sequences (GGA) in the 160 NT 5’-UTR of the *cspA* transcript (data not shown) and an RNA-seq analysis conducted previously did not reveal *cspA* mRNA among the 721 different transcripts that copurified with CsrA [2].

Based on all of these observations, we conclude that, under the growth conditions tested, the BarA-UvrY TCS directly exerts its global effect on gene expression primarily through the Csr system by activating the transcription of the CsrB and CsrC sRNAs, with *cspA* representing a likely exception. This raises the question of why UvrY-FLAG crosslinked *in vivo* (though weakly) to the regulatory regions of 286 genes *in E*. *coli* and 301 genes in *Salmonella* (S2 Table), but exerted little or no regulatory effect in the examples that were tested. To explain these observations, we propose three possible hypotheses: (i) Perhaps other unknown activator(s) are required along with UvrY/SirA for the expression of these genes, but were not available or functioning under the chosen growth conditions. Thus, UvrY/SirA might activate some of these genes under other growth conditions. (ii) Perhaps one or more repressors overrides the influence of UvrY/SirA on the expression of these genes under the conditions tested [62]. To address these two hypotheses, we searched for factors known to regulate the expression of the putative UvrY target genes. This analysis revealed several DNA binding proteins including CRP, FNR and IHF appear to regulate the greatest number of potential UvrY targets (S7 Fig). Hence, it is conceivable that such factors may mask the effects of UvrY on the expression of these genes under our growth conditions. (iii) Because the putative UvrY targets from the ChIP lack the 18nt-long IR sequence found in the regulatory regions of *csrB/C*, it is also possible that some of these genes have degenerate UvrY/SirA binding sites with no functional relevance or perhaps serving as nonspecific holding sites for UvrY/SirA [62, 63].

### UvrY expression is activated by CsrA via a putative posttranscriptional mechanism that does not involve the RNA helicase DeaD, a known regulator of *uvrY* translation

**Fig 9:**
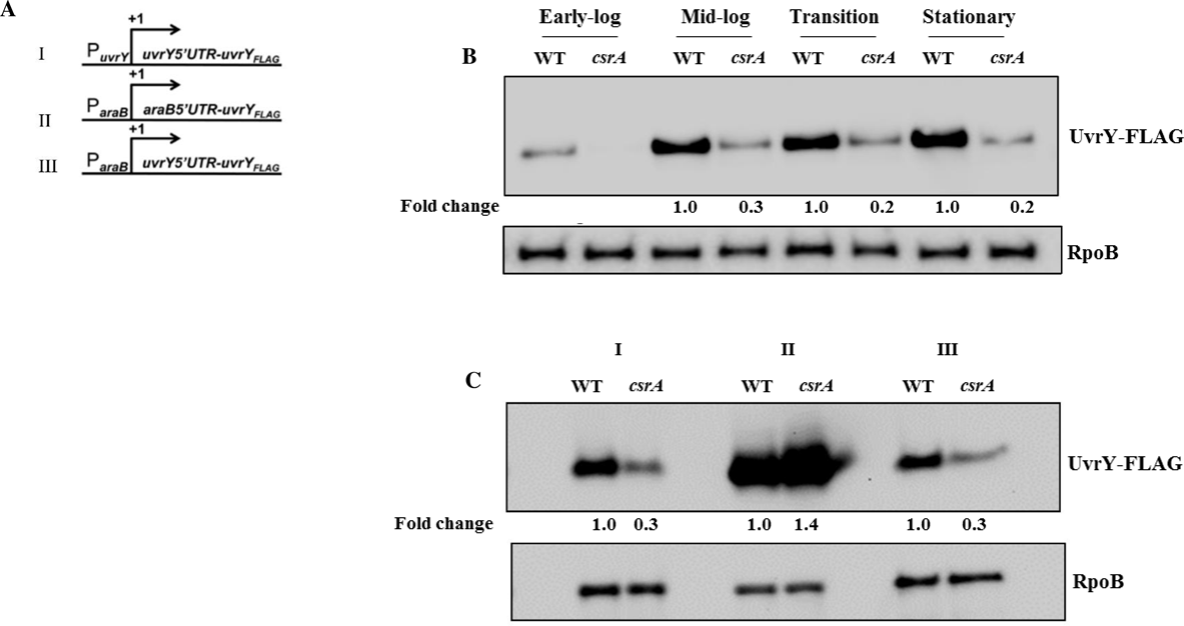
Effect of CsrA on *uvrY* expression. Gene fusions used in this study: (i) native *uvrY* regulatory region and coding sequence containing a FLAG^®^ tag; (ii) *araB* promoter and mRNA nocoding leader fused to the *uvrY-FLAG* coding sequence and (iii) *araB* promoter fused at the transcription start site to the *uvrY* noncoding leader and *uvrY-FLAG* coding sequence (A) as previously depicted (4). Effects of *csrA* disruption on expression of the native *uvrY-FLAG* construct (panel A, i) in LB at different growth phases (B). Effects of *csrA* disruption on expression of the *uvrY-FLAG* constructs shown in panel A (C). Strains were grown in the presence of arabinose, and UvrY-FLAG was detected at mid-log phase of growth by Western blotting. RpoB served as a loading control for these experiments.

In addition to BarA-UvrY, the transcription of CsrB/C is also strongly activated by CsrA [13, 16, 35] by mechanisms that are yet to be determined. Recent evidence suggested that CsrA positively affects *uvrY* expression both at the transcriptional and translational levels [35]. Moreover, CsrA is required for switching BarA from a protein possessing phosphatase activity to kinase activity on UvrY [35]. Thus, we tested the effect of CsrA on the expression, stability and phosphorylation of UvrY-FLAG. The results showed that while CsrA does not affect UvrY-FLAG protein stability (S8A Fig), it has a strong positive effect on UvrY-FLAG expression (Fig 9B). We observed a 5-fold reduction in UvrY-FLAG levels in a *csrA*::*kan* mutant strain compared to the isogenic wild type strain. These results confirm that CsrA regulates the expression of CsrB and CsrC by activating UvrY expression. Moreover, *in silico* analyses did not reveal any typical CsrA binding sites in the 5’-UTR of the *uvrY* transcript (data not shown) and *uvrY* mRNA was not among the 721 different transcripts that co-purified with CsrA [2], suggesting that the CsrA effect on *uvrY* expression is likely to be indirect.

**Fig 10:**
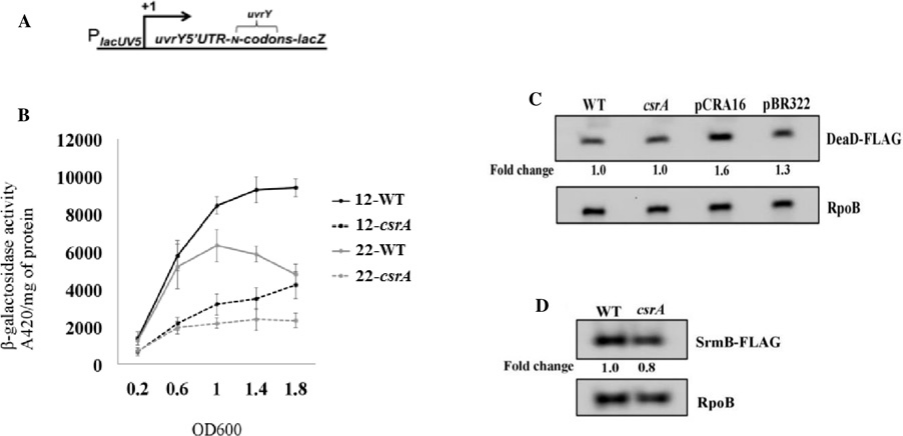
CsrA activates *uvrY* expression without affecting DeaD or SrmB RNA helicase levels. The *uvrY* gene fusions used in this study were previously depicted in (4): *lacUV5* promoter fused at the transcription start site to the *uvrY* mRNA leader and N (12 or 22) *uvrY* codons fused in frame to *lacZ* (A). The effect of *csrA* disruption on expression of P*lacUV5*-*uvrY’–’lacZ* reporter fusions is shown (B). Cells were grown in LB and harvested at various times throughout growth and assayed for β-galactosidase specific activity (A_420_/mg protein). The values represent the average of two independent experiments. Error bars depict standard error of the means. Western blots showing effects of *csrA* on the level of DeaD-FLAG (C) or SrmB-FLAG (D) in an MG1655 derivative that expresses the corresponding FLAG-tagged gene from its native genomic locus are shown. Effect of *csrA*::*kan* complemented by a *csrA* expression plasmid (pCRA16) or with a control plasmid (pBR322) is also shown for DEAD-FLAG. RpoB served as a loading control for these blots.

To assess the genetic effects of CsrA on UvrY expression, we analyzed fusion derivatives that replaced the *uvrY* promoter or the promoter and noncoding leader with those from *araB* (Fig 9A) [4]. Results from these experiments showed that regulation by CsrA requires the *uvrY* noncoding leader but not the promoter region (Fig 9C). To test whether CsrA requires sequence from the *uvrY* coding region for regulation, we measured the expression of a *uvrY’-’lacZ* translational reporter fusion containing the leader and 12 or 22 codons of *uvrY* in wild type and *csrA*::*kan* strains (Fig 10A). The two different leaders were chosen because previous studies showed that the DeaD RNA helicase is needed to override inhibitory long-distance mRNA base-pairing between the *uvrY* noncoding leader and the proximal *uvrY* coding segment, and that DeaD regulates *uvrY* expression from gene fusions with 22 or more *uvrY* codons, but not from fusions with less than this, e.g. 12 codons, which do not permit the inhibitory base-pairing [4]. Our results showed that expression of both fusions (22 and 12 codon) was decreased in the *csrA*::*kan* strain by 2.8 and 2.6-fold, respectively (Fig 10B), suggesting that the effects of CsrA are not mediated via DeaD. We also tested the effect of CsrA on the expression of a DeaD-FLAG fusion, expressed from the native chromosomal locus, and confirmed that CsrA does not regulate *deaD* expression (Fig 10C).

Furthermore, another RNA helicase, SrmB, was shown to regulate the transcription of CsrB and CsrC RNAs by an undefined mechanism, which is different than that of DeaD [4]. We, therefore, examined the effect of CsrA on the levels of SrmB-FLAG and observed no effect on the expression of this RNA helicase (Fig 10D). Thus, CsrA does not appear to regulate expression of either of the DeaD-box RNA helicases that activate CsrB and CsrC transcription.

Finally, in a screen to find novel direct CsrA targets by RNAseq analysis, *ihfA* mRNA was found to co-purify with CsrA [2], suggesting that CsrA might regulate production of the IHF protein. Therefore, we hypothesized that in addition to activating *uvrY* expression, CsrA might also activate the expression of *csrB* by activating the expression of IHF. However, we observed no effect of CsrA on IhfA-FLAG or IhfB-FLAG levels when these fusions were expressed from their native chromosomal loci (S8B Fig). Altogether, our results indicate that CsrA activates *csrB/C* transcription by activating *uvrY* expression, most likely through a posttranscriptional mechanism that is yet to be defined.

### Effects of ppGpp and DksA, mediators of the stringent response, on *csrB* expression

**Fig 11:**
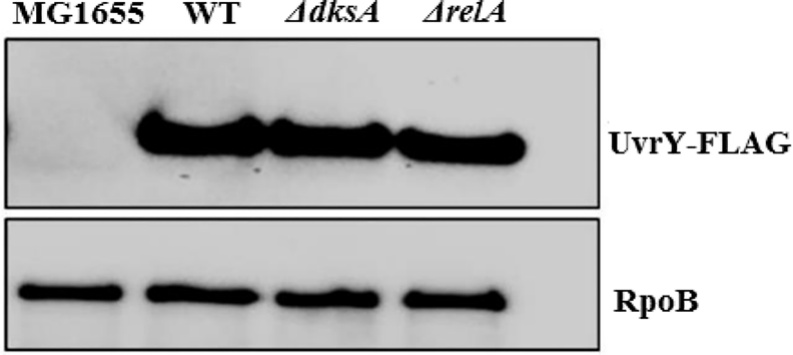
Effect of DksA and RelA on UvrY-FLAG levels. Western blotting of UvrY-FLAG levels in strains MG1655 (no FLAG fusion), WT (MG1655 expressing UvrY-FLAG from the *uvrY* genomic locus), and isogenic ∆*dksA* and ∆*relA* strains. Proteins were collected from cultures grown in LB medium to mid-exponential growth phase (~OD_600_ of 0.6). RpoB served as a loading control.

**Fig 12:**
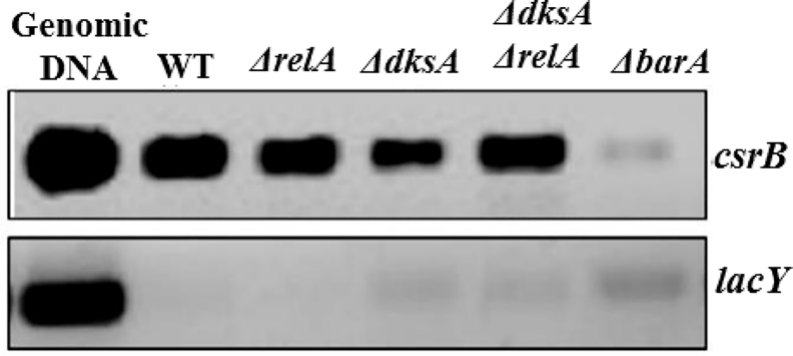
Effect of DksA and RelA on *in vivo* binding of UvrY to *csrB* promoter. The effect of DksA and RelA on *in vivo* binding of UvrY to *csrB* promoter was determined by ChIP-PCR assay in a WT (MG1655 expressing UvrY-FLAG) and isogenic ∆*dksA*, ∆*relA* and ∆*barA* strains. Agarose gel showing PCR amplification of *csrB* promoter region recovered from each strain. The *lacY* gene served as a negative control in this experiment.

**Fig 13:**
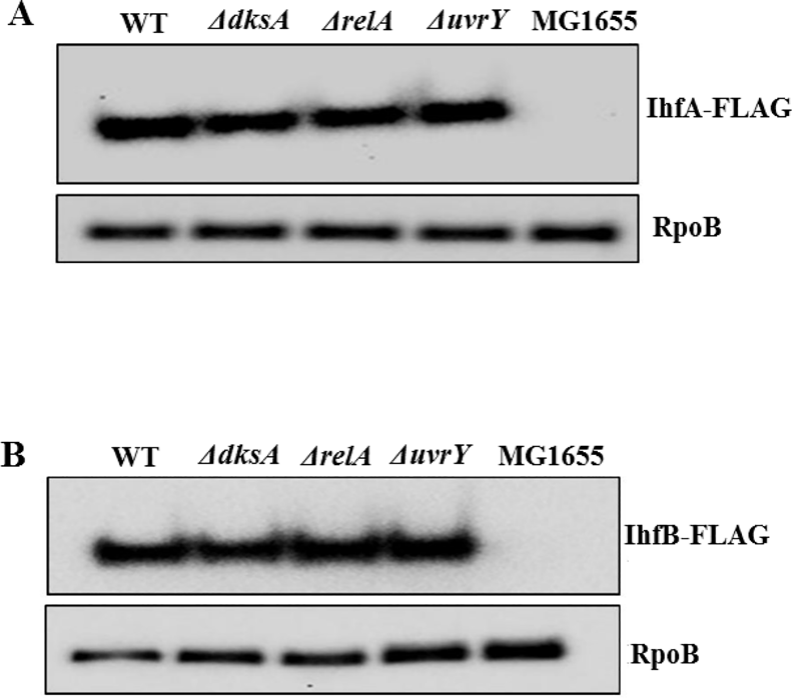
Effect of DksA, RelA and UvrY on the expression of IHF subunits. Western blotting of IhfA-FLAG (A) and IhfB-FLAG (B) proteins examined in WT (MG1655 expressing *ihfB-FLAG* or *ihfA-FLAG* fusions from the native genomic loci) and an MG1655 control lacking the *FLAG* fusions. Proteins from isogenic strains with ∆*dksA*, ∆*relA* and ∆*uvrY* disruption are as shown. Proteins were collected from cultures grown in LB medium to mid-exponential growth phase (~OD_600_ of 0.6). RpoB loading controls for these analyses are also shown.

Previously, ppGpp and DksA were shown to strongly activate expression of CsrB and CsrC in *E*. *coli* [2]. However, the mechanism for these effects is yet to be elucidated. In an attempt to define this mechanism, we hypothesized that ppGpp and DksA might activate the expression of factor(s) that are known to activate CsrB/C transcription i.e., UvrY, IHF, CsrA, DeaD and/or SrmB. Previous studies showed that ppGpp and DksA positively affect CsrA levels, but this effect appears to be too modest to account for their strong effects on CsrB/C levels [2]. Therefore, we first determined whether DksA and ppGpp affect the *in vivo* levels of UvrY-FLAG protein (Western blot) and the *in vivo csrB* DNA binding (ChIP-PCR) by UvrY in Δ*dksA*, Δ*relA* (encoding the major ppGpp synthase) and isogenic wild type strains. It was found that ppGpp and DksA had weak or negligible effects on UvrY levels (Fig 11), *in vivo* binding of UvrY to *csrB* (Fig 12), and the *in vivo* phosphorylation status of the UvrY protein (S3 Fig). Next, we tested the effects of DksA and ppGpp on IHF expression. The results showed no effect of DksA or ppGpp on IhfA-FLAG (Fig 13A) or IhfB-FLAG levels under our growth conditions (Fig 13B).

**Fig 14:**
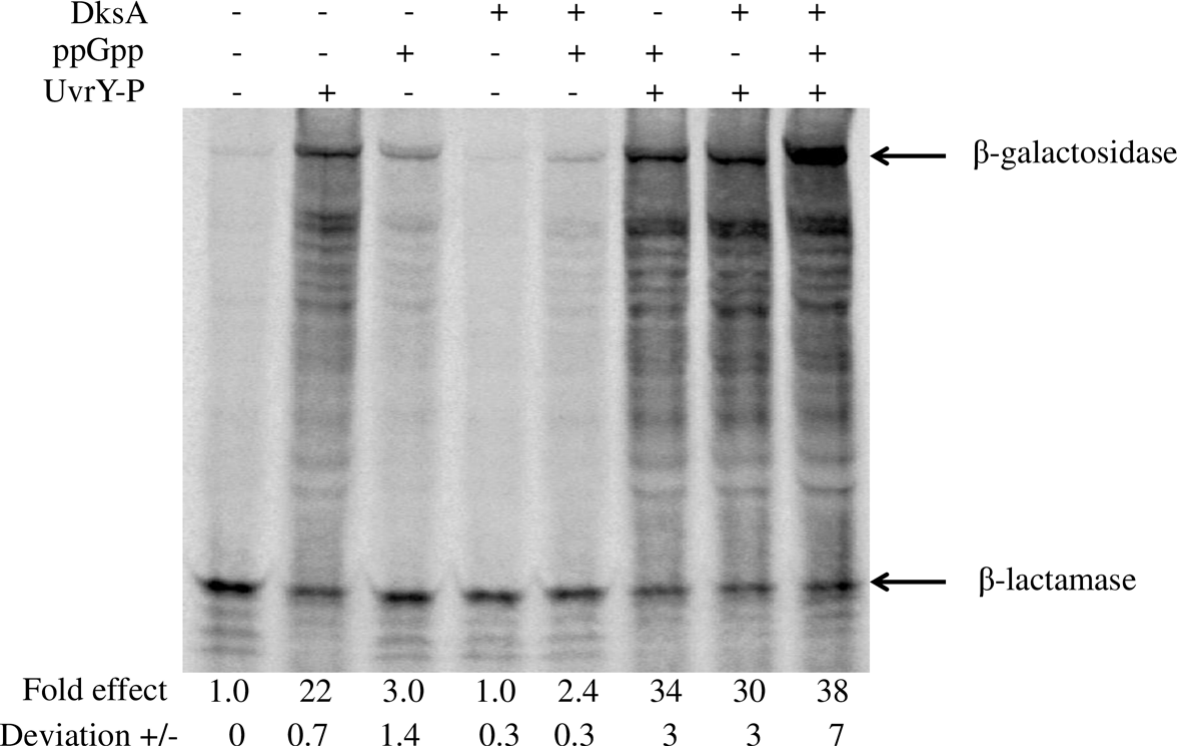
*In vitro* transcription-translation of a supercoiled plasmid-encoded *csrB-lacZ* transcriptional fusion. Reactions contained pLFXcsrB-lacZ plasmid (2 μg), UvrY-P (2.3 μM), ppGpp (250 μM) and/or DksA (2 μM) as indicated. Incorporation of ^35^S-labeled methionine into protein products was detected by SDS PAGE followed by phosphorimaging. Signal intensity of the full length protein was determined using Quantity One software. The fold-effects of regulatory factors were determined with respect to the control reaction lacking the factors, after normalization against the internal control, β-lactamase, which was encoded on the same plasmid. Absolute deviation for each reaction was determined from two independent experiments.

Because ppGpp and DksA did not substantially affect the *in vivo* expression of IHF or alter the *in vivo* binding of UvrY-P to *csrB* DNA, we decided to test their direct effects on the *in vitro* transcription of *csrB* and *csrC* genes. However, neither gene was expressed in defined transcription reactions that contained UvrY-P and IHF in the case of *csrB* and basal reaction components (5nM RNAP; 40 mM Tris·HCl, pH 7.9; NaCl (165 mM); 5% glycerol; 10 mM MgCl_2_; 1 mM DTT; 0.1 μg/μl BSA; 500 μMATP; 200 μM CTP and UTP; 10 μMGTP and [α-32P] GTP (2.5 μCi). This suggests that unknown factors may be required for *csrB/C* transcription (data not shown). We next used coupled transcription-translation in S-30 extracts to determine whether the addition of ppGpp and DksA would directly regulate expression of *csrB/C* in the presence of other cellular factors present in the S-30 extracts but not the defined transcription reactions. As previously reported [13, 16], UvrY-P strongly stimulated the expression of the full-length β-galactosidase from *csrB-lacZ* (Fig 14) and *csrC-lacZ* (S9 Fig) transcriptional fusions in this assay. A series of truncated β-galactosidase products was also observed in these reactions, which quantitatively responded to activators similarly to the full-length protein. While expression in the absence of UvrY-P was weak, the addition of ppGpp or ppGpp and DksA to the UvrY-deficient *csrB-lacZ* reaction caused a modest increase in expression (Fig 14). In the presence of UvrY-P, addition of ppGpp alone modestly activated *csrB-lacZ* expression (1.5-fold). DksA alone also had weak effects in the presence of UvrY-P (1.4-fold), while the addition of both ppGpp and DksA led to a 1.7-fold increase in *csrB-lacZ* expression. Addition of ppGpp alone, in the absence of UvrY-P, resulted in a slight increase in *csrC-lacZ* expression (S9 Fig). However, ppGpp and/or DksA failed to activate *csrC-lacZ* expression in reactions containing UvrY-P. We conclude that ppGpp and DksA directly activate *csrB* expression, at least partially accounting for the stimulatory effect of these regulators on the *in vivo* expression of this gene [2]. However, ppGpp and/or DksA may be indirectly involved in or require a factor that was deficient in our assays for *csrC* expression.

As mentioned above, DeaD and SrmB activate *csrB* and *csrC* transcription by distinct mechanisms [4]. While DeaD activates *uvrY* translation, the mechanism by which SrmB activates *csrB/C* transcription is still not defined. Our ChIP-PCR data revealed that SrmB is required for normal binding of UvrY to *csrB in vivo* (S2 Fig). This effect of SrmB on UvrY binding occurs without it altering UvrY or UvrY-P levels [4]. Because neither ppGpp nor DksA had substantial effects on *in vivo* binding of UvrY to *csrB* DNA (Fig 12) we infer that the mechanism by which ppGpp and DksA activate *csrB/C* transcription does not involve SrmB and vice versa.

### *In silico* analysis of other possible targets of UvrY (SirA) binding

In many cases, transcription factors compete for binding to their DNA binding sites with other transcription factors, which may play antagonistic roles in the regulation of the target gene(s) [64]. Hence, we reasoned that there could be additional targets of UvrY/SirA in the genomes of *E*. *coli* / *Salmonella* that were not captured by ChIP-exo. To test this hypothesis, we performed *in silico* analysis using the *Ab Initio* Motif Identification Environment (AIMIE) database [65]. We scanned the *E*. *coli* genome using the first six bases of the 18 bp (TGTAAGNNNNNNCTTACA) UvrY binding sequence, followed by manually checking the presence of the rest of the IR DNA sequence in the regulatory region of each discovered putative target. In this way, we identified 19 putative target genes containing the 18-bp UvrY-P IR sequence either perfectly or near perfectly conserved in their regulatory regions (S10 Fig). Next, we searched for factors known to directly regulate the expression of these putative targets and compared the respective DNA binding motif of each factor with the 18 bp IR UvrY binding motif. Out of the 19 putative targets, regulatory factors were previously established for 5 of them (S11 Fig). When we compared the respective DNA binding motifs of each factor with the IR binding motif of UvrY, we found an overlap in all of them (S11 Fig), supporting the possibility that undiscovered direct target sequences for UvrY binding might have gone undetected in our experiments. Whether these putative target sequences function in UvrY regulation under other growth conditions will require future investigation.

### Phylogenetic distribution of BarA, UvrY, CsrA and FliW

**Fig 15:**
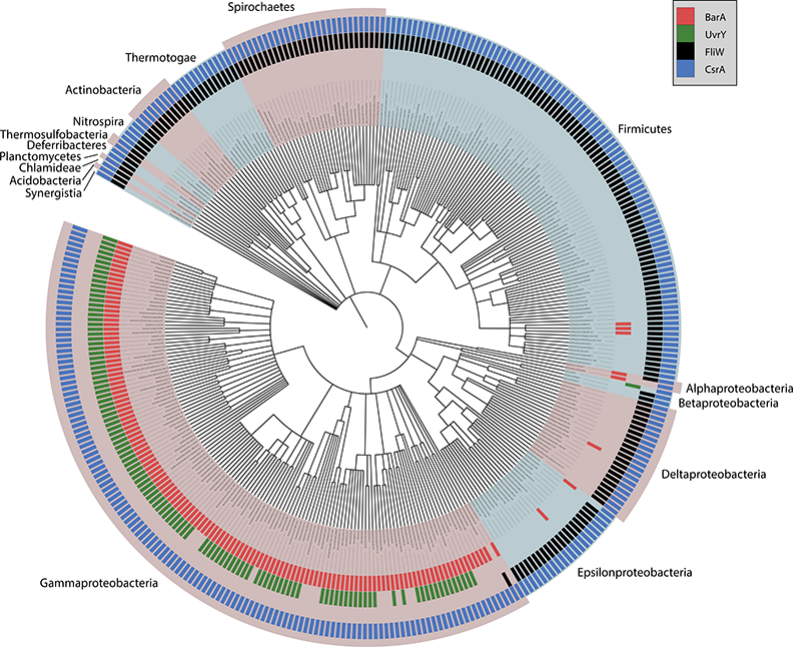
The distribution of CsrA, BarA, UvrY and FliW across phylogenies indicates a strong anti-correlation between BarA/UvrY and FliW. Depicted is a reference phylogeny of fully-sequenced genomes encoding CsrA and at least one of BarA, UvrY or FliW, plotting their presence/absence data obtained from orthology databases. The results show that the presence of BarA-UvrY and FliW are significantly anti-correlated.

The BarA-UvrY TCS exerts global effects on gene expression by activating CsrB and CsrC transcription, thus controlling CsrA activity [16, 35]. This signaling pathway is common to the commensal bacterium *E*. *coli*, the pathogen *Salmonella*, as well as a variety of other γ-proteobacterial pathogens [66]. In contrast, little is known about the workings of the Csr system in other species that possess *csrA* homologs, but have not been shown to express CsrA-inhibitory sRNAs. Recently, Mukherjee et al. have shown that the FliW protein of *B*. *subtilis* binds to CsrA and antagonizes its activity, thus preventing it from binding to the flagellin mRNA, *hag* [41, 67]. Because BarA-UvrY is devoted to transcription of CsrB/C sRNAs in *E*. *coli* and *Salmonella* and *fliW* is absent in species known to produce CsrA-inhibitory sRNAs [41], we hypothesized that BarA-UvrY and FliW might represent different modules for regulating CsrA activity in different bacterial species. To test this hypothesis, we examined the phylogenetic distributions of CsrA, BarA-UvrY and FliW across fully-sequenced bacteria (S3 Table). We found that, for species encoding at least one readily identifiable CsrA ortholog, the presence of BarA-UvrY and FliW were strongly anti-correlated at species level (Spearman correlation = -0.97, p = 1.10x10^-205^) and genus level (Spearman correlation = -0.95, p = 7.31x10^-74^) (Fig 15). Of the 346 genomes encoding CsrA, 340 also encoded either BarA-UvrY or FliW, but not both, and only 6 species might encode both BarA-UvrY and FliW systems: *Desulfosporosinus acidiphilus*, *D*. *meridiei*, *D*. *orientis*, *D*. *baculatum*, *Magnetococcus marinus*, and *Candidatus Sulfuricurvum sp* (Fig 15 and S3 Table). Interestingly, in all cases where both BarA/UvrY and FliW may function, only the BarA component appears to be present, which may indicate that this two-component system has lost or is losing its function in these species. Additional experimental data are needed in order to confirm this hypothesis.

Accurate and complete identification of orthologs across a broad taxonomic range is a challenging problem, with potential for both false-positives and false-negatives [68]. Although difficult to detect, any strong biases in the methodology we used to identify orthologous genes could affect our results. To address this concern, we used two additional and widely-used methods to identify alternative ortholog sets: KEGG orthology database (KO) [53], and UniProt reference clusters of orthologs (UniRef) [54]. We found that, regardless of the method used to identify orthologs, FliW and BarA-UvrY were strongly negatively correlated, particularly when conditioning on the presence of CsrA (S3 Table). In addition, we applied sequence similarity filtering and manual curation to reduce false positives and false negatives in the NCBI orthologs data set.

After the sequence similarity filtering and manual curation, we found several false positives for CsrA in the Streptococcus genus, such as misannotated heavy metal stress response proteins (NCBI identification numbers (GI): 15675047, 13622200, 21904470), peptide methionine sulfoxide reductases (GI: 222114035, 134272076, 209540564, 24638057, 342165139, 342165138), and putative CsrA homologs that lack the N-terminal and C-terminal conserved regions of CsrA (GI: 895760047, 882844105, 882819224). Most of the main false negatives that we identified are BarA and UvrY from genomes that match the representative homologs from *E*. *coli* within the similarity and coverage thresholds (see methods) such as *Pseudomonas* (NCBI genome accession numbers: NC_022594, NC_022591, NC_022361, NC_022360), *Xanthomonas* (NC_020815, NC_017271, NC_017267, NC_016010, NC_013722), *Pseudoxanthomonas* (NC_014924), *Shewanella* (NC_009052, NC_009665, NC_008321, NC_008322, NC_017566, NC_016901), and other members of the γ-Proteobacteria class (S3 Table).

These false positives and false negatives caused a small increase of 2% in the negative correlation, reinforcing the present conclusions (Spearman correlation = -0.95 vs. -0.97, comparing the unfiltered and filtered data sets, respectively) (S3 Table). These results argue against methodological bias as strongly affecting our results. Together, these results indicate that the negative correlation between FliW and BarA-UvrY regulatory systems is highly unlikely to be artifactual and thus represents a biologically relevant observation. The implications of this observation for the possible regulation of CsrA activity by inhibitory sRNAs in FliW-encoding species will require additional investigation to unravel.

## Conclusions

Using ChIP-exo [34], we probed the complete repertoire of UvrY (SirA) DNA binding sites in the genomes of *E*. *coli* and *Salmonella*. We discovered that the *csrB*/C genes are by far the strongest direct targets of UvrY in these species. UvrY binds specifically to an 18 nt palindromic sequence in the promoter regions of *csrB/C* and exhibited an almost absolute requirement for phosphorylation by BarA for this binding *in vivo* under growth conditions examined. UvrY-P requires IHF for optimal binding to and activation of *csrB* but not *csrC*. CsrA activates *csrB/C* transcription by activating *uvrY* expression by an undefined mechanism, which may require the noncoding mRNA leader, but does not involve the other known posttranscriptional regulator of *uvrY*, the DeaD-box RNA helicase DeaD [4]. The RNA DEAD-box helicase SrmB, which also activates *csrB/C* transcription in *E*. *coli* [4], promoted binding of UvrY to *csrB* DNA *in vivo* without affecting the expression of other factors known to activate *csrB* expression. This suggests that SrmB may regulate unknown factor(s) involved in *csrB* transcription. The stringent response factors ppGpp and DksA activate CsrB/C expression *in vivo* [2] and were found to modestly activate *csrB* expression *in vitro* in S-30 extracts. Whether this involves direct binding to RNA polymerase remains to be determined.

Genomic loci that crosslinked weakly to UvrY were identified proximal to the promoter regions of 286 genes in *E*. *coli* and 301 genes in *Salmonella*, respectively (S2 Table). However, further analysis showed weak or negligible regulatory effects of UvrY on the expression of the genes that were tested (S6A–S6C Fig). Hence, we conclude that, under the growth conditions that we have examined, UvrY-P exerts its global effects on gene expression almost entirely by activating the transcription of CsrB and CsrC. We suspect that most of the genes that have been found to respond to BarA and UvrY in *E*. *coli* [31] and their orthologs in other species [30, 32] are indirect targets of UvrY, which are regulated by CsrA.

The BarA-UvrY/Csr signaling pathway has been studied in the commensal bacterium *E*. *coli*, the pathogen *Salmonella*, and a number of other γ-proteobacterial pathogens [66]. *B*. *subtilis*, the only Gram-positive bacterium in which CsrA has been studied to date, uses FliW as an α-CsrA protein, which binds to and inhibits CsrA activity [41, 67]. While this is only the first such example, FliW is present in diverse species, where it may act as an inhibitor of CsrA. For species encoding at least one readily identifiable CsrA ortholog, the presence of BarA-UvrY and FliW were strongly anti-correlated. This suggests that while γ*-*Proteobacteria use the BarA-UvrY TCS to control CsrA by activating the transcription of its sRNA antagonists, members of the β-Proteobacteria, δ-Proteobacteria, ε-Proteobacteria, Firmicutes, Spirochaetes, Thermotogae, Actinobacteria, Nitrospira, Thermosulfobacteria, Deferribacteres, Planctomycetes, Chlamideae, Acidobacteria, and Synergistia may use FliW to regulate CsrA activity.

## Supporting Information

S1 Fig SirA-FLAG and UvrY-His_6_ are functional *in vivo*. Western blot showing expression of SirA-FLAG (A) and Northern blots showing CsrB levels in 14028S (wild type *Salmonella*), 14028S strain with *sirA-FLAG* fusion integrated at the native *sirA* locus and *sirA* and *csrB* deletion strains (B). CsrB levels in MG1655, *uvrY* deletion and UvrY-His_6_ (expressed from pET24-a expression vector) *E*. *coli* strains (C). For Western blotting, RpoB loading controls shown (A). For Northern blotting, the 16S/23S rRNA loading controls are shown. Cultures were grown in LB to mid-exponential growth phase (OD_600_ of 0.6).
(TIFF)

S2 Fig ChIP specificity confirmation by PCR. Polymerase chain reaction was used to confirm the specificity of ChIP assay. Primers (S1 Table) annealing to the promoter regions of *csrB*, *lacY* and 16S rDNA (*rrsH*) were used to amplify the promoters of *csrB*, *lacY* and/or 16S rDNA genes from DNA that was crosslinked and immunoprecipitated from *E*. *coli* (panel A) or *Salmonella* (panel B). In these analysis, *csrB* was used as a positive control and *lacY* and 16S rDNA (*rrsH*) were used as negative controls for *E*. *coli* and *Salmonella*, respectively.
(TIFF)

S3 Fig Effect of BarA, IHF, DksA, RelA and CsrA on UvrY-FLAG and UvrY-FLAG-P levels. Phos-tag SDS-PAGE with Western blotting was used for detection of the phosphorylated (P-UvrY-FLAG) and non-phosphorylated (UvrY-FLAG) protein levels expressed in a WT (MG1655 expressing UvrY-FLAG) and isogenic ∆*barA*, ∆*dksA*, ∆*relA*, ∆*ihfA*, ∆*ihfB*, *csrA*::*kan and* ∆*uvrY* strain. Cultures were grown in LB to mid-exponential growth phase (OD_600_ of 0.6). The relative levels and % of phosphorylation of UvrY in the WT, *barA*, *dksA*, *relA*, *ihfA*, *ihfB* and *csrA* are: 1.0, 1.2, 1.0, 1.0, 1.2, 1.2, 0.23 (UvrY levels) and 7%, 1%, 6.8%, 6.9%, 9%, 9% and 8% (% of UvrY phosphorylation), respectively.
(TIFF)

S4 Fig DNase I footprinting of *csrC* DNA by phosphorylated and non-phosphorylated UvrY. A ^32^P-end labeled DNA probe (reverse strand) that included both the upstream and downstream putative UvrY binding sites was used (Fig 1E). Reactions in all lanes except lanes 1 contained DNase I (0.025U/12.5ul reaction). Reactions in lanes 3–6 and lanes 7–10 contained 0.25, 0.35, 0.5, 0.7 μM of phosphorylated and non-phosphorylated UvrY-His_6_, respectively. Lane 2 reaction contained no UvrY.
(TIFF)

S5 Fig Effect of IHF on UvrY-FLAG levels. Western blotting of UvrY-FLAG levels in strains MG1655 (no FLAG fusion), WT (MG1655 expressing UvrY-FLAG), and isogenic ∆*ihfA* and ∆*ihfB* strains. Cultures were grown in LB to mid-exponential growth phase (OD_600_ of 0.6). RpoB loading control is also shown.
(TIFF)

S6 Fig Examination of putative UvrY targets discovered by ChIP-exo. Northern blot showing effect of UvrY and Csr factors on SpoT42 sRNA (A). Cultures of MG1655 and the isogenic mutants indicated were grown in Kornberg medium containing 0.5% glucose, to early stationary growth phase (OD_600_ of 2.0). The 16S/23S rRNA loading controls are also shown. Western blot showing effect of *uvrY* deletion on FhuF-FLAG protein (B). Cultures were grown in LB to mid-exponential growth phase (OD_600_ of 0.6), at which point dipyridyl was added to culture (1mM final concentration). Samples were collected before and 10 min after the addition of dipyridyl. RpoB loading control is also shown. Electrophoretic gel mobility shift assay showing UvrY binding to *cspA*, *spf*, *fhuF* and *csrB* DNA (C). Phosphorylated (UvrY-P) UvrY-His_6_ binding to *spf*, *cspA*, *fhuF*, *csrB* and *rrlE* DNA was tested by EMSA as shown. The *spf*, *cspA*, *fhuF* and *csrB* DNA probes used in this experiments encompass the ChIP-exo derived putative UvrY binding sites discovered in the promoter region of each gene (shown in Fig 1 and S2 Table). The DNA probes (0.5 nM) were incubated at room temperature with increasing concentration of *in vitro* phosphorylated UvrY-His_6_ protein. End-labeled 0.5 nM *rrlE* and 50-fold cold *csrB* (for the specific competitor, marked with *, 0.65 μM UvrY-P was used) were also used as non-specific and specific competitors, respectively. The DNA-protein complexes were resolved in a non-denaturing 7% polyacrylamide gel. Shifted protein-DNA complex is indicated in black arrows. Effect of UvrY on the expression of putative sRNA targets (D). Northern blots showing effect of UvrY on the expression of several sRNA genes. Cultures were grown in Kornberg, supplemented with 0.5% glucose, to mid-exponential (OD_600_ of 0.6), transition to stationary (OD_600_ of 1.2) and stationary growth phases (OD_600_ of 3.0). The 5S rRNA loading control is also shown.
(TIF)

S7 Fig List of transcription factors known to regulate expression of the ChIP-exo derived putative UvrY targets (listed in S2 Table). (TIFF)

S8 Fig Effect of CsrA on UvrY-FLAG protein stability and expression of IHF subunits. Western blot UvrY-FLAG protein stability (A) in MG1655 (no FLAG fusion), WT (MG1655 with a *uvrY-FLAG* fusion integrated at the *uvrY* locus) and isogenic *csrA* mutant. Cells were grown in LB to mid-exponential growth phase (OD_600_ of 0.6) at which point tetracycline and chloramphenicol were added and cultures were sampled thereafter at the times shown. Western blotting of IhfA-FLAG and IhfB-FLAG proteins (B) examined in MG1655 or an isogenic *csrA*::*kan* mutant expressing *ihfA-FLAG* or *ihfB-FLAG* fusions from the native genomic loci (WT). Cultures were grown in LB to mid-exponential growth phase (~OD_600_ of 0.6). RpoB loading controls for these analyses are also shown.
(TIFF)

S9 Fig *In vitro* transcription-translation of a supercoiled plasmid-encoded *csrC-lacZ* fusion. Reactions contained pLFXcsrC-lacZ (4 μg), UvrY-P (2.3 μM), ppGpp (250 μM) and/or DksA (2 μM) as indicated. Incorporation of ^35^S-labeled methionine into protein products was detected by SDS PAGE with phosphorimaging. Signal intensity was determined using Quantity One software. The fold-effects of regulatory factors were determined with respect to the control reaction lacking these factors, after normalization against β-lactamase as an internal control. Absolute deviation was determined from two independent experiments.
(TIFF)

S10 Fig Putative UvrY targets derived by *in silico* analysis. 19 Putative UvrY targets were derived by *in silico* analysis using the *Ab Initio* Motif Identification Environment (AIMIE) database (46). This was done by scanning the *E*. *coli* genome using the first six bases of the 18bp-long (TGTAAGNNNNNNCTTACA) UvrY consensus binding sequence, followed by manually checking the presence of the rest of the IR DNA sequence in the regulatory region of each discovered putative target. The predicted UvrY binding sequence in the promoter region of each putative target in comparison to the UvrY consensus sequence is shown. Distance from the center of the predicted UvrY binding sequence to the known transcription start site (TSS) of each putative target is shown. Nucleotides marked in red are the mismatch between the UvrY consensus sequence and the predicted UvrY binding sequence.
(TIFF)

S11 Fig Overlap between predicted UvrY binding site and consensus DNA binding site of known regulators of putative UvrY targets derived by *in silico* analysis. Shown in this figure are five of the 19 putative UvrY targets identified by *in silico* analysis (S9 Fig), for which regulatory factors were previously established. The list of the five putative targets, their respective known regulators, the consensus DNA binding site of each regulator (capitalized) and the overlap between the consensus binding site of the known regulator and the predicted UvrY binding site within the promoter of each putative target (underlined) is shown.
(TIFF)

S1 Table List of strains, plasmids, bacteriophages and primers used in this study (DOCX)

S2 Table List of putative UvrY-SirA targets identified by ChIP-exo. (XLSX)

S3 Table Distribution of BarA, UvrY, CsrA, and FliW at species and genus level. We identified CsrA, BarA, UvrY and FliW orthologs from all fully-sequenced bacterial genomes in the NCBI genomes database (http://www.ncbi.nlm.nih.gov/genome/). The results were compared to other orthology databases and filtered by sequence similarity and alignment coverage in order to minimize the number of false positives/negatives (see Methods). At both species and genus levels, the results exhibited a strong anti-correlation between BarA-UvrY and FliW.
(XLSX)
