# Supplementary figures and images for: Genomic Targets and Features of BarA-UvrY (-SirA) Signal Transduction Systems
Source: PLoS One. 2015 Dec 16;10(12):e0145035. doi: 10.1371/journal.pone.0145035 (PMC4682653; doi:10.1371/journal.pone.0145035)

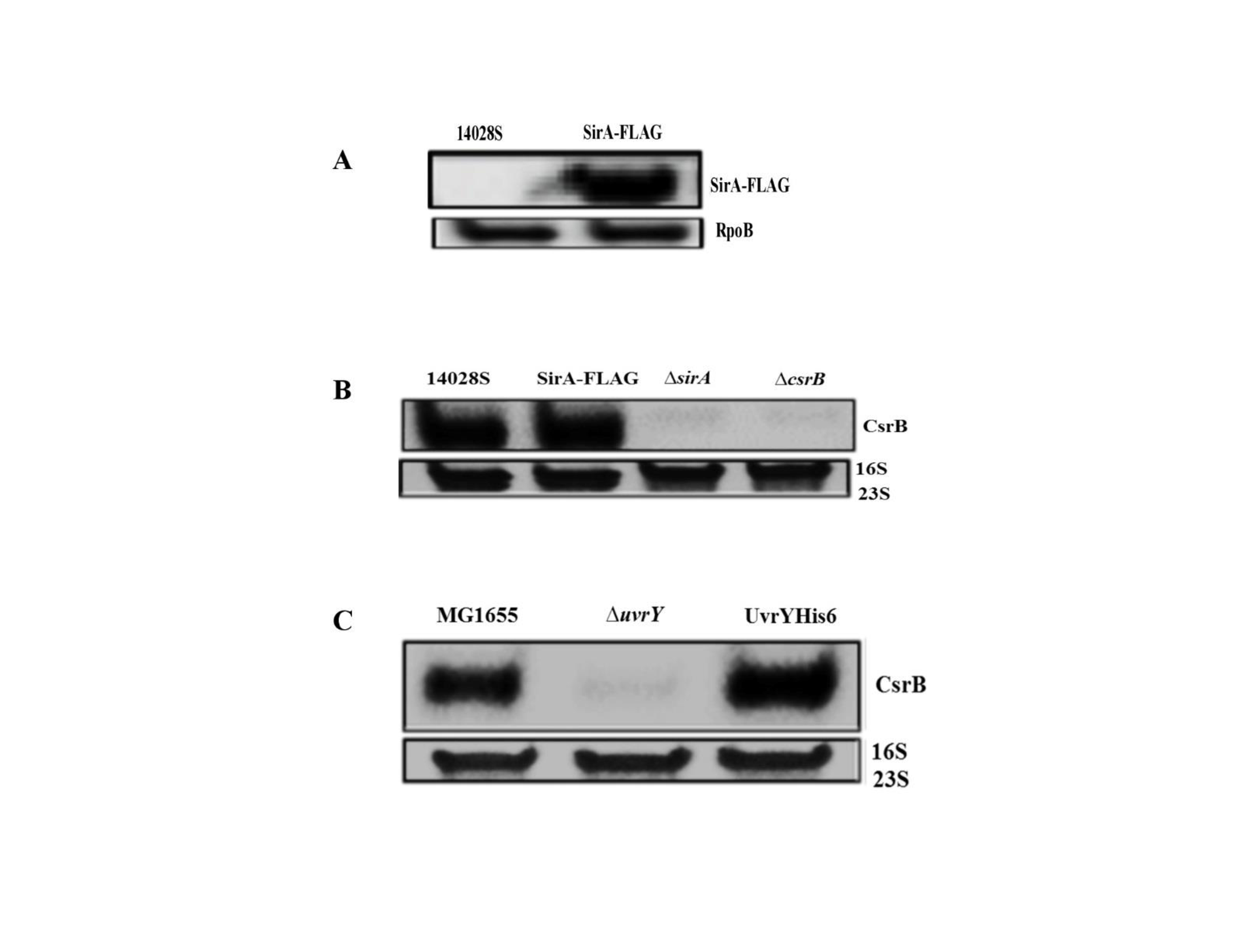

Supplement: S1 Fig — Western blot showing expression of SirA-FLAG (A) and Northern blots showing CsrB levels in 14028S (wild type Salmonella), 14028S strain with sirA-FLAG fusion integrated at the native sirA locus and sirA and csrB deletion strains (B). CsrB levels in MG1655, uvrY deletion and UvrY-His6 (expressed from pET24-a expression vector) E. coli strains (C). For Western blotting, RpoB loading controls shown (A). For Northern blotting, the 16S/23S rRNA loading controls are shown. Cultures were grown in LB to mid-exponential growth phase (OD600 of 0.6). (TIFF) [file pone.0145035.s001.tiff]

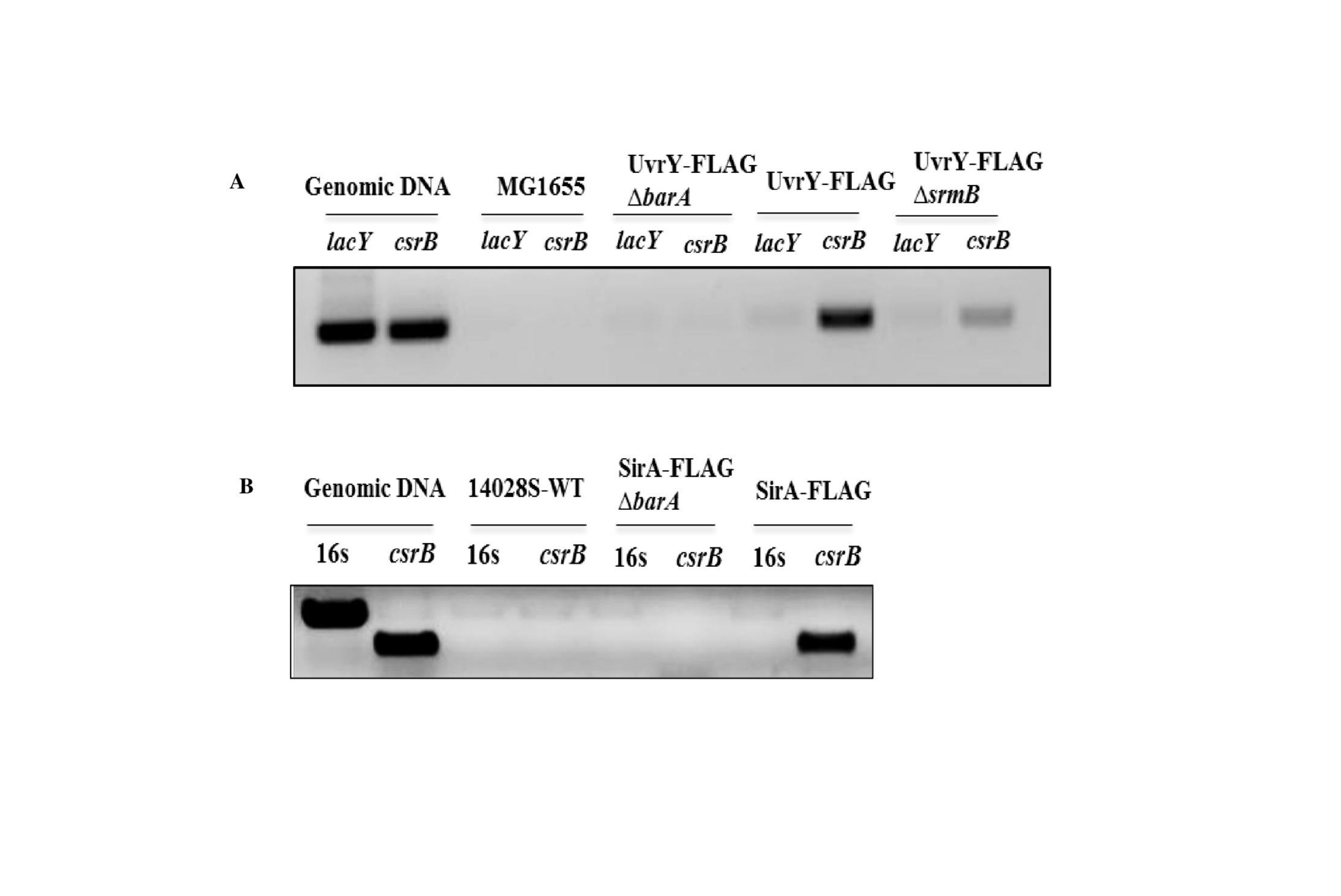

Supplement: S2 Fig — Polymerase chain reaction was used to confirm the specificity of ChIP assay. Primers (S1 Table) annealing to the promoter regions of csrB, lacY and 16S rDNA (rrsH) were used to amplify the promoters of csrB, lacY and/or 16S rDNA genes from DNA that was crosslinked and immunoprecipitated from E. coli (panel A) or Salmonella (panel B). In these analysis, csrB was used as a positive control and lacY and 16S rDNA (rrsH) were used as negative controls for E. coli and Salmonella, respectively. (TIFF) [file pone.0145035.s002.tiff]

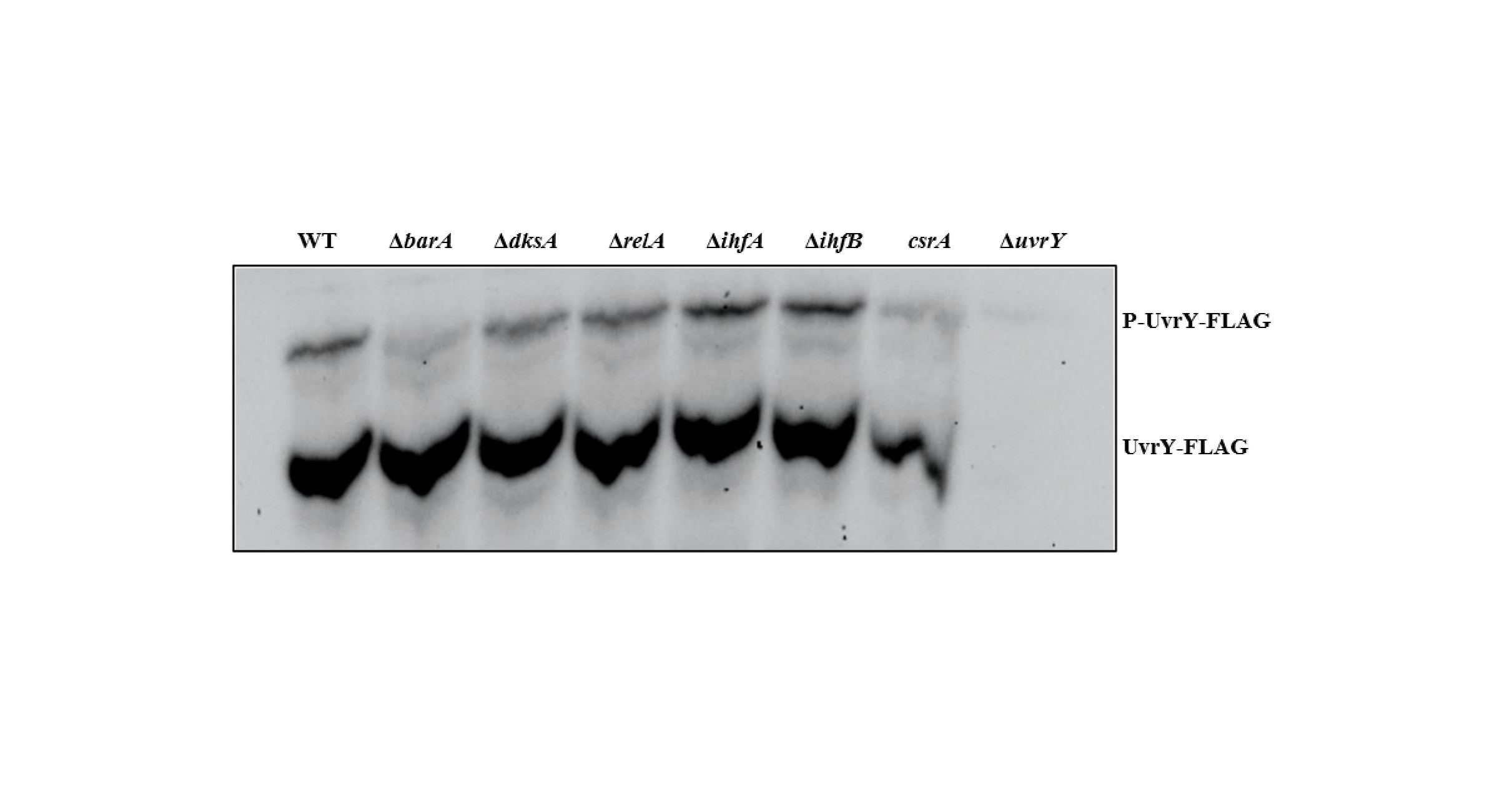

Supplement: S3 Fig — Phos-tag SDS-PAGE with Western blotting was used for detection of the phosphorylated (P-UvrY-FLAG) and non-phosphorylated (UvrY-FLAG) protein levels expressed in a WT (MG1655 expressing UvrY-FLAG) and isogenic ∆barA, ∆dksA, ∆relA, ∆ihfA, ∆ihfB, csrA::kan and ∆uvrY strain. Cultures were grown in LB to mid-exponential growth phase (OD600 of 0.6). The relative levels and % of phosphorylation of UvrY in the WT, barA, dksA, relA, ihfA, ihfB and csrA are: 1.0, 1.2, 1.0, 1.0, 1.2, 1.2, 0.23 (UvrY levels) and 7%, 1%, 6.8%, 6.9%, 9%, 9% and 8% (% of UvrY phosphorylation), respectively. (TIFF) [file pone.0145035.s003.tiff]

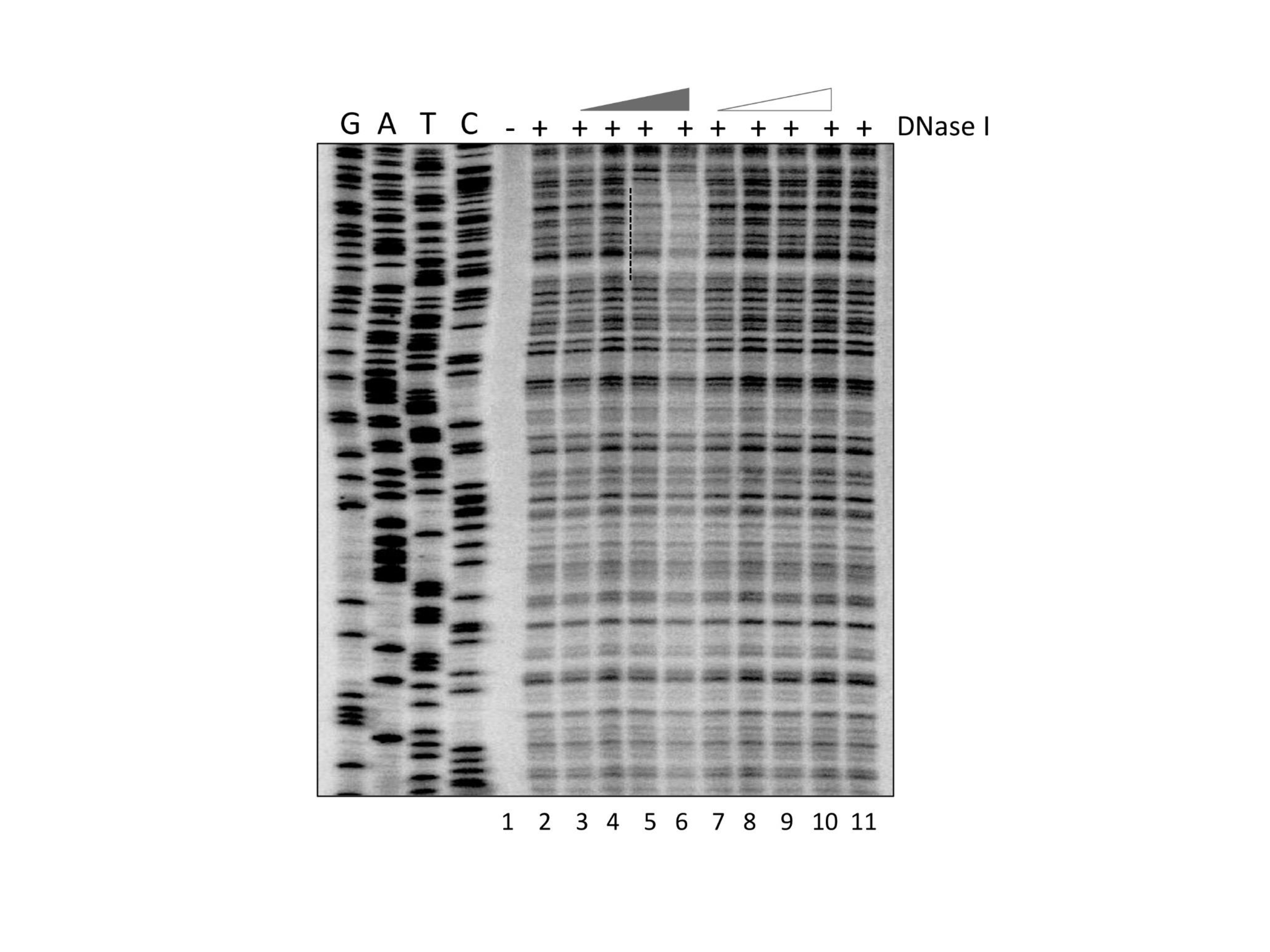

Supplement: S4 Fig — A 32P-end labeled DNA probe (reverse strand) that included both the upstream and downstream putative UvrY binding sites was used (Fig 1E). Reactions in all lanes except lanes 1 contained DNase I (0.025U/12.5ul reaction). Reactions in lanes 3–6 and lanes 7–10 contained 0.25, 0.35, 0.5, 0.7 μM of phosphorylated and non-phosphorylated UvrY-His6, respectively. Lane 2 reaction contained no UvrY. (TIFF) [file pone.0145035.s004.tiff]

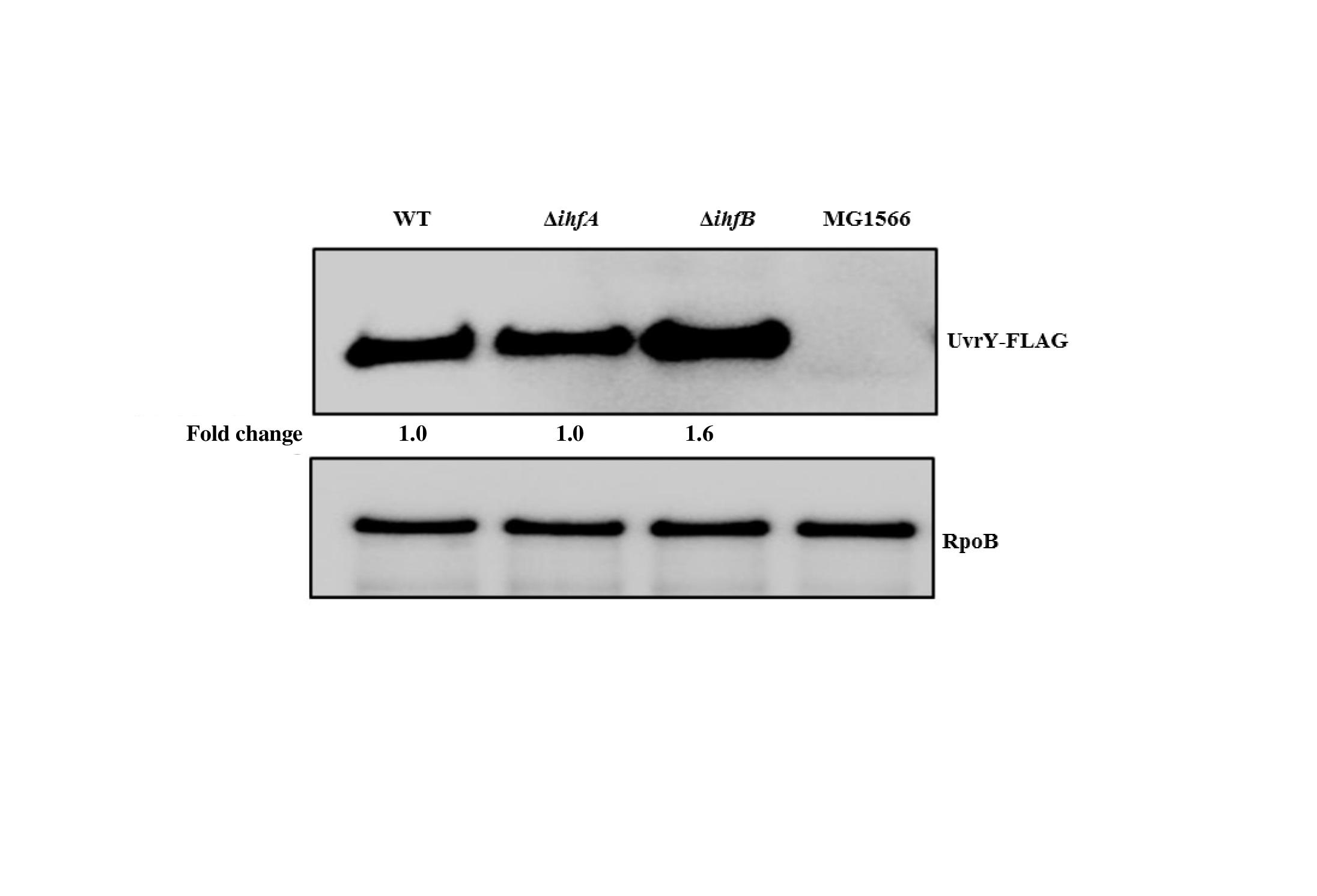

Supplement: S5 Fig — Western blotting of UvrY-FLAG levels in strains MG1655 (no FLAG fusion), WT (MG1655 expressing UvrY-FLAG), and isogenic ∆ihfA and ∆ihfB strains. Cultures were grown in LB to mid-exponential growth phase (OD600 of 0.6). RpoB loading control is also shown. (TIFF) [file pone.0145035.s005.tiff]

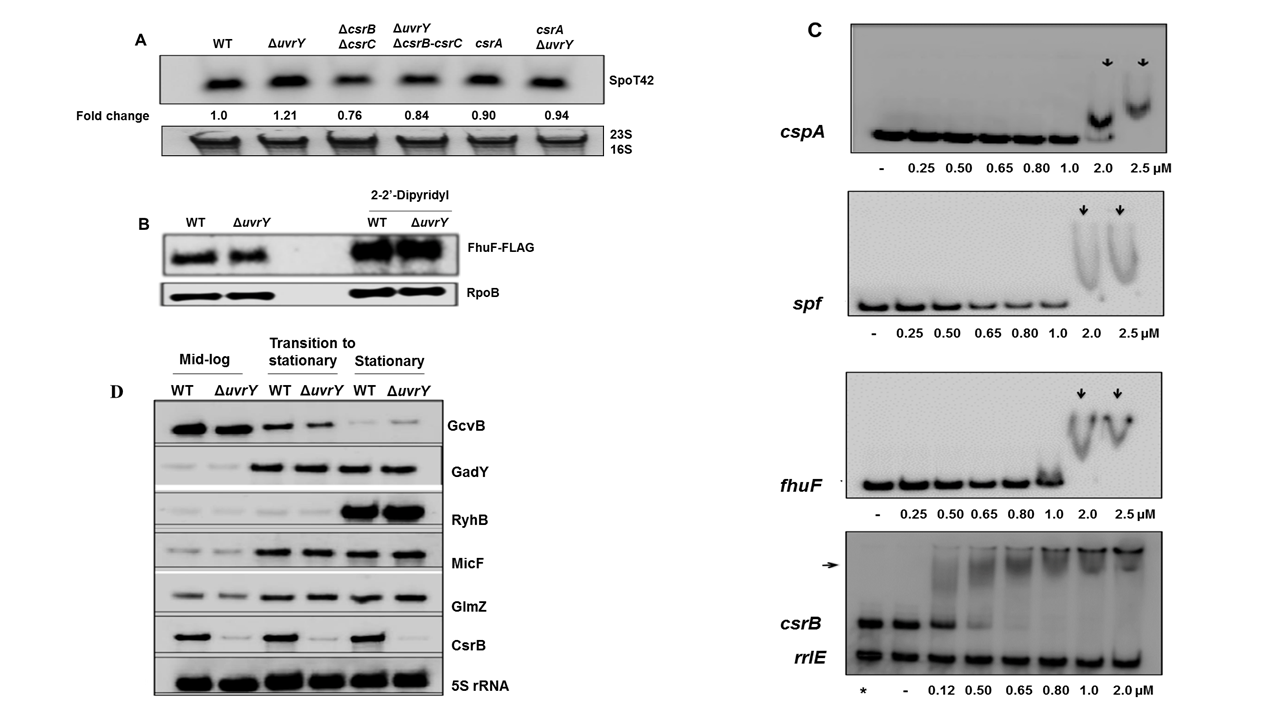

Supplement: S6 Fig — Northern blot showing effect of UvrY and Csr factors on SpoT42 sRNA (A). Cultures of MG1655 and the isogenic mutants indicated were grown in Kornberg medium containing 0.5% glucose, to early stationary growth phase (OD600 of 2.0). The 16S/23S rRNA loading controls are also shown. Western blot showing effect of uvrY deletion on FhuF-FLAG protein (B). Cultures were grown in LB to mid-exponential growth phase (OD600 of 0.6), at which point dipyridyl was added to culture (1mM final concentration). Samples were collected before and 10 min after the addition of dipyridyl. RpoB loading control is also shown. Electrophoretic gel mobility shift assay showing UvrY binding to cspA, spf, fhuF and csrB DNA (C). Phosphorylated (UvrY-P) UvrY-His6 binding to spf, cspA, fhuF, csrB and rrlE DNA was tested by EMSA as shown. The spf, cspA, fhuF and csrB DNA probes used in this experiments encompass the ChIP-exo derived putative UvrY binding sites discovered in the promoter region of each gene (shown in Fig 1 and S2 Table). The DNA probes (0.5 nM) were incubated at room temperature with increasing concentration of in vitro phosphorylated UvrY-His6 protein. End-labeled 0.5 nM rrlE and 50-fold cold csrB (for the specific competitor, marked with *, 0.65 μM UvrY-P was used) were also used as non-specific and specific competitors, respectively. The DNA-protein complexes were resolved in a non-denaturing 7% polyacrylamide gel. Shifted protein-DNA complex is indicated in black arrows. Effect of UvrY on the expression of putative sRNA targets (D). Northern blots showing effect of UvrY on the expression of several sRNA genes. Cultures were grown in Kornberg, supplemented with 0.5% glucose, to mid-exponential (OD600 of 0.6), transition to stationary (OD600 of 1.2) and stationary growth phases (OD600 of 3.0). The 5S rRNA loading control is also shown. (TIF) [file pone.0145035.s006.tif]

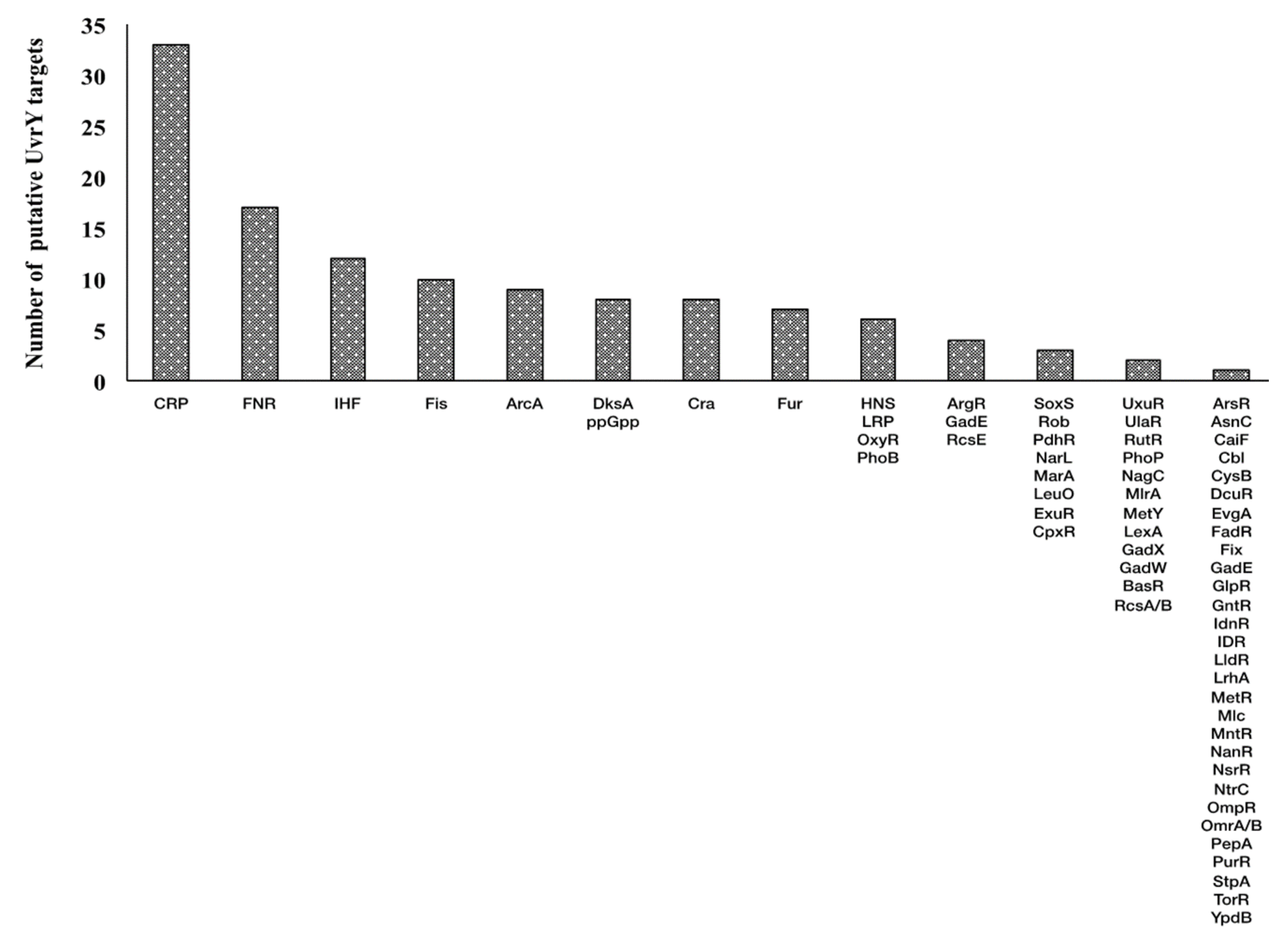

Supplement: S7 Fig — (TIFF) [file pone.0145035.s007.tiff]

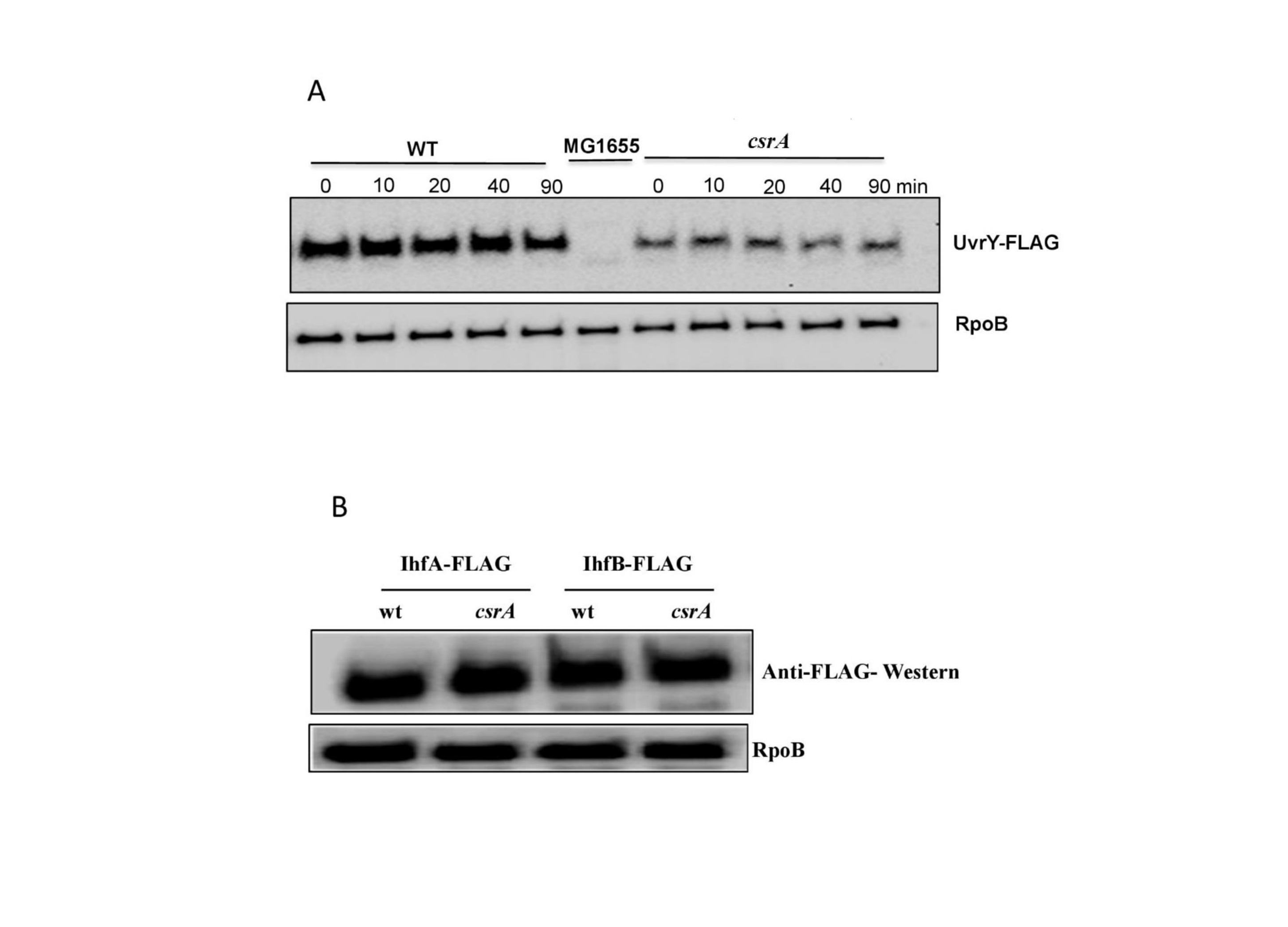

Supplement: S8 Fig — Western blot UvrY-FLAG protein stability (A) in MG1655 (no FLAG fusion), WT (MG1655 with a uvrY-FLAG fusion integrated at the uvrY locus) and isogenic csrA mutant. Cells were grown in LB to mid-exponential growth phase (OD600 of 0.6) at which point tetracycline and chloramphenicol were added and cultures were sampled thereafter at the times shown. Western blotting of IhfA-FLAG and IhfB-FLAG proteins (B) examined in MG1655 or an isogenic csrA::kan mutant expressing ihfA-FLAG or ihfB-FLAG fusions from the native genomic loci (WT). Cultures were grown in LB to mid-exponential growth phase (~OD600 of 0.6). RpoB loading controls for these analyses are also shown. (TIFF) [file pone.0145035.s008.tiff]

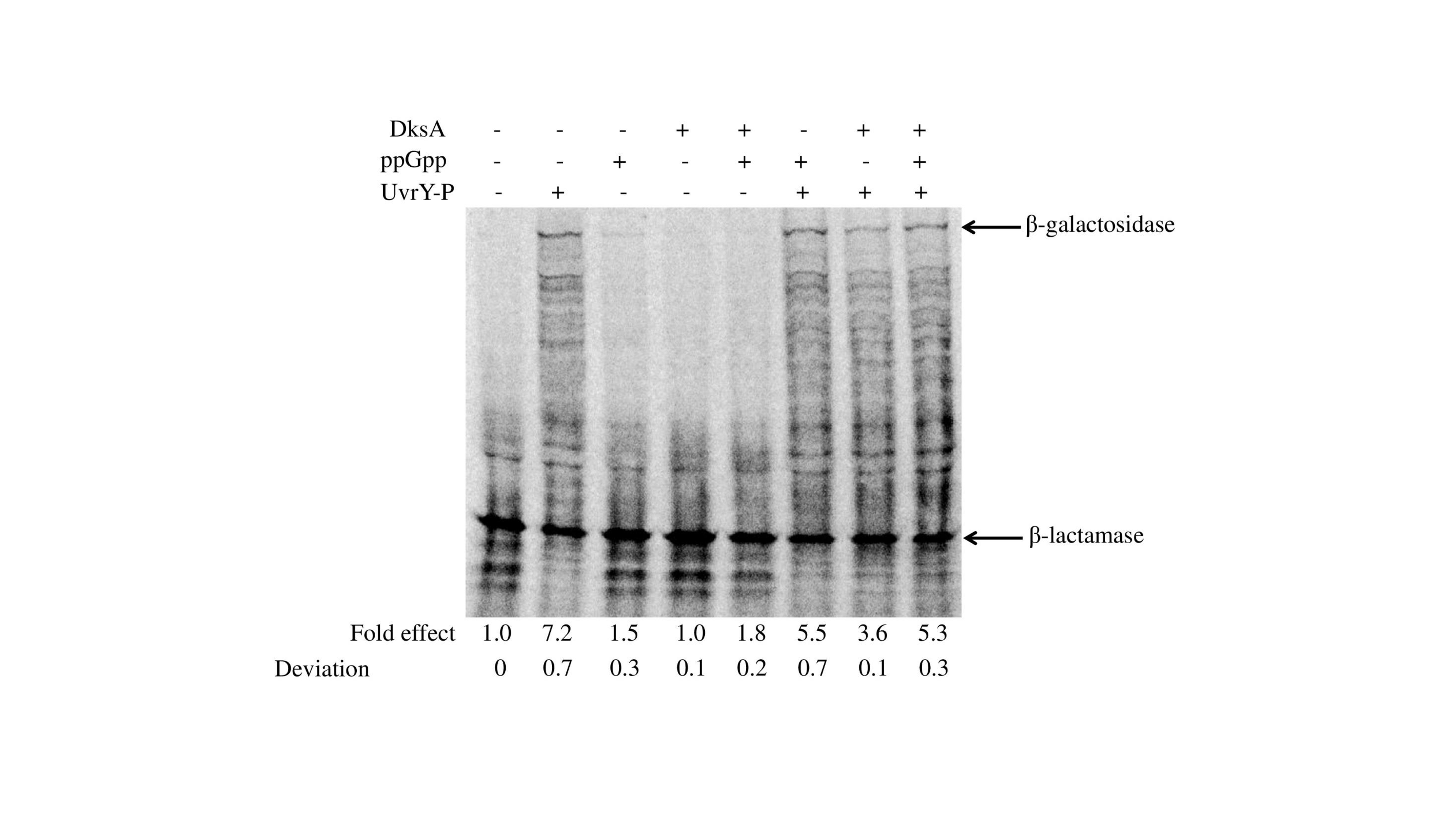

Supplement: S9 Fig — Reactions contained pLFXcsrC-lacZ (4 μg), UvrY-P (2.3 μM), ppGpp (250 μM) and/or DksA (2 μM) as indicated. Incorporation of 35S-labeled methionine into protein products was detected by SDS PAGE with phosphorimaging. Signal intensity was determined using Quantity One software. The fold-effects of regulatory factors were determined with respect to the control reaction lacking these factors, after normalization against β-lactamase as an internal control. Absolute deviation was determined from two independent experiments. (TIFF) [file pone.0145035.s009.tiff]

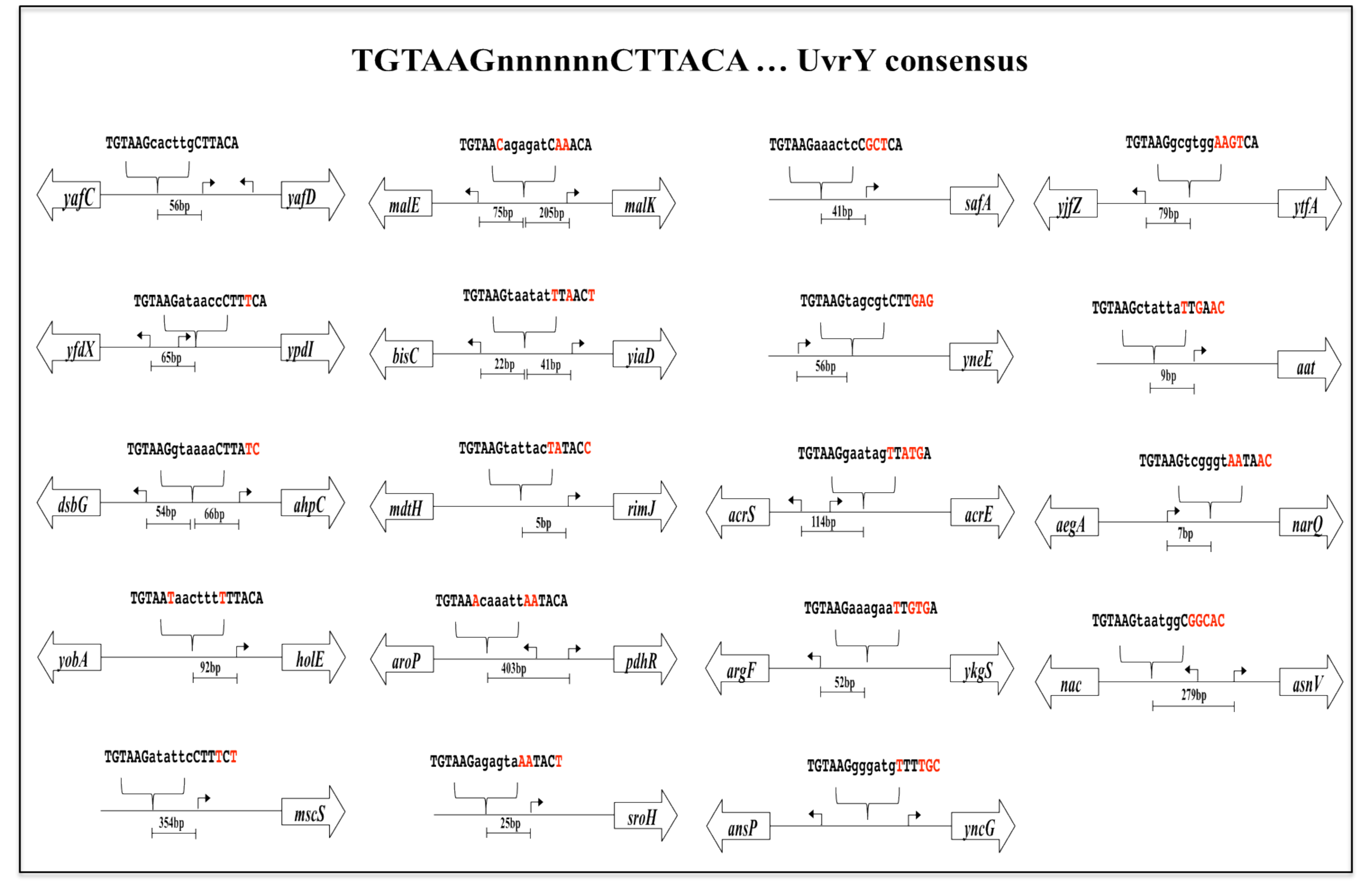

Supplement: S10 Fig — 19 Putative UvrY targets were derived by in silico analysis using the Ab Initio Motif Identification Environment (AIMIE) database (46). This was done by scanning the E. coli genome using the first six bases of the 18bp-long (TGTAAGNNNNNNCTTACA) UvrY consensus binding sequence, followed by manually checking the presence of the rest of the IR DNA sequence in the regulatory region of each discovered putative target. The predicted UvrY binding sequence in the promoter region of each putative target in comparison to the UvrY consensus sequence is shown. Distance from the center of the predicted UvrY binding sequence to the known transcription start site (TSS) of each putative target is shown. Nucleotides marked in red are the mismatch between the UvrY consensus sequence and the predicted UvrY binding sequence. (TIFF) [file pone.0145035.s010.tiff]

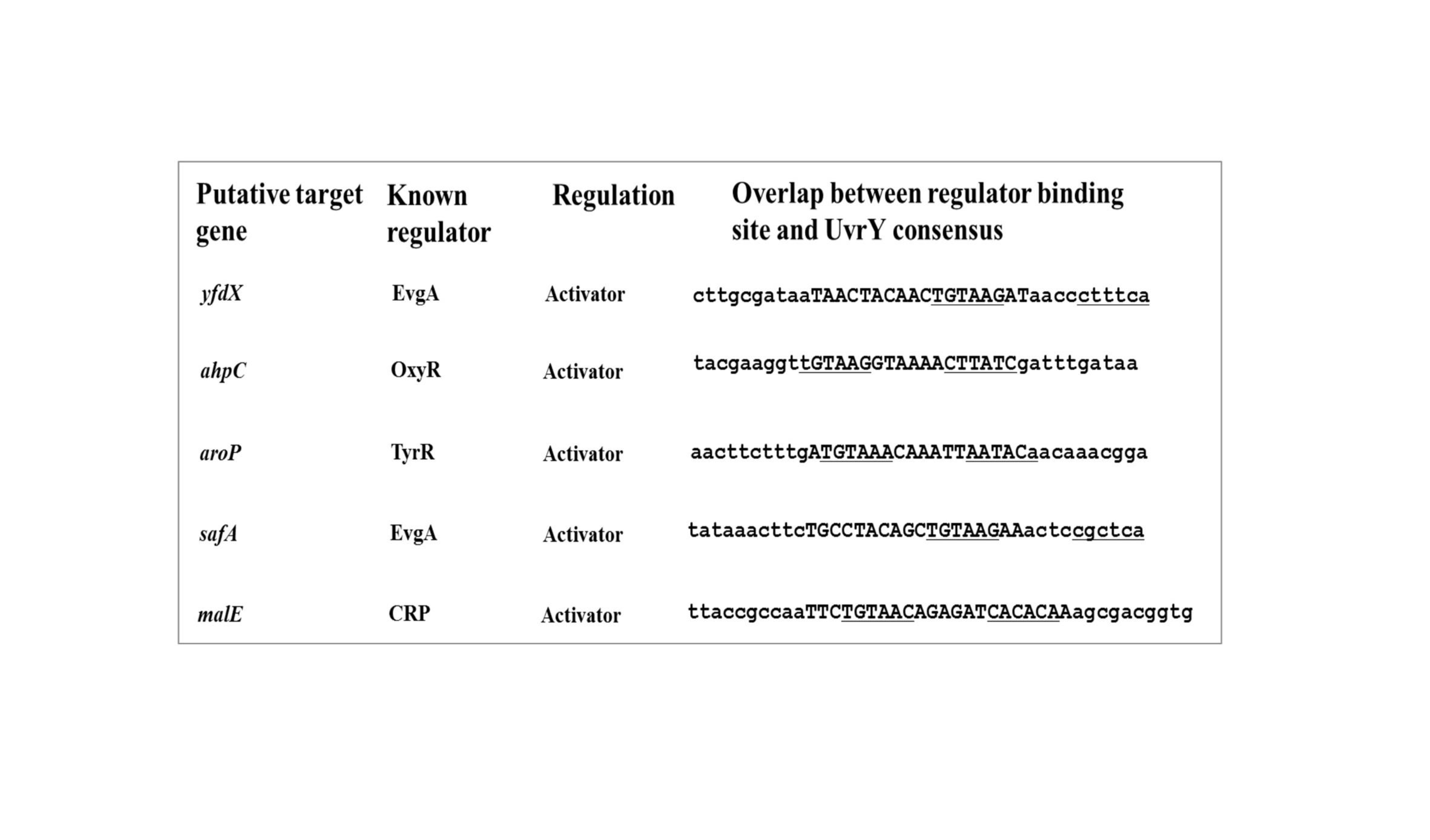

Supplement: S11 Fig — Shown in this figure are five of the 19 putative UvrY targets identified by in silico analysis (S9 Fig), for which regulatory factors were previously established. The list of the five putative targets, their respective known regulators, the consensus DNA binding site of each regulator (capitalized) and the overlap between the consensus binding site of the known regulator and the predicted UvrY binding site within the promoter of each putative target (underlined) is shown. (TIFF) [file pone.0145035.s011.tiff]
