# Supplementary material for: Genomic Targets and Features of BarA-UvrY (-SirA) Signal Transduction Systems
Source: PLoS One. 2015 Dec 16;10(12):e0145035. doi: 10.1371/journal.pone.0145035 (PMC4682653; doi:10.1371/journal.pone.0145035)
Supplement: S1 Table — (DOCX) [file pone.0145035.s012.docx]

**Table 1:** List of strains, plasmids and bacteriophages used

| **Strain** | **Description** | **Reference** |
| --- | --- | --- |
| MG1655 | Prototrophic *E. coli* K12 | Michael Cashel |
| MG1655 ∆*barA* | MG1655 with unmarked *barA* mutation | (1) |
| MG1655 *csrA*::*kan* | MG1655 with *csrA*::*kan^r^* | (2) |
| MG1655 ∆*uvrY* | MG1655 with *uvrY::cam* | (3) |
| MG1655 ∆*csrB* | MG1655 with marked *csrB* deletion - *Kan^r^* | This study |
| MG1655 ∆*csrC* | MG1655 with marked *csrC* deletion - *Kan^r^* | This study |
| MG1655 ∆*uvrY csrA*::*kan* | *uvrY*_::_*cam* allele introduced by transduction using P1*vir* | This study |
| MG1655 ∆*uvrY* ∆*ihfA* | MG1655 ∆*uvrY with marked ihfA deletion -Kan^r^* | This study |
| MG1655 ∆*uvrY* ∆*ihfB* | MG1655 ∆*uvrY with marked ihfB deletion -Kan^r^* | This study |
| MG1655 ∆*csrB* ∆*csrC* | MG1655 with marked *csrB (Cam^r^)* and *csrC deletions (Tet^r^)* deletions. | This study |
| MG1655 ∆*csrB* ∆*csrC* ∆*uvrY* | *uvrY*_::_*kan* allele introduced by transduction using P1*vir* | This study |
| MG1655 *uvrY*_FLAG_ | MG1655 with in-frame CTD 3X –FLAG tag at native *uvrY* locus - *kan*^r^ | (3) |
| MG1655 *csrA*::*kan* *uvrY*_FLAG_ | *csrA::kan* allele introduced by transduction using P1*vir* | This study |
| MG1655 ∆*barA* *uvrY*_FLAG_ | MG1655-*uvrY*_FLAG_ with unmarked *barA* deletion | This study |
| MG1655 ∆*ihfA* *uvrY*_FLAG_ | MG1655-*uvrY*_FLAG_ with unmarked *ihfA* deletion | This study |
| MG1655 ∆*ihfB uvrY*_FLAG_ | MG1655-*uvrY*_FLAG_ with unmarked *ihfB* deletion | This study |
| MG1655 ∆*ihfA* ∆*ihfB uvrY*_FLAG_ | MG1655-*uvrY*_FLAG_ with unmarked *ihfA* and *ihfB* double deletions | This study |
| MG1655 ∆*relA uvrY*_FLAG_ | *relA::kan* allele introduced by transduction using P1*vir* | This study |
| MG1655 ∆*dksA uvrY*_FLAG_ | *dksA::tet* allele introduced by transduction using P1*vir* | This study |
| MG1655 ∆*dksA* ∆*relA uvrY*_FLAG_ | MG1655-*uvrY*_FLAG_ with *dksA* and *relA* double deletions | This study |
| MG1655 ∆*srmB uvrY*_FLAG_ | MG1655-*uvrY*_FLAG_ with *srmB* deletion | (3) |
| MG1655 *ihfA*_FLAG_ | MG1655 with in-frame CTD 3X –FLAG tag at native *ihfA* locus - *Kan*^r^ | This study |
| MG1655 ∆*relA ihfA*_FLAG_ | *relA::kan* allele introduced by transduction using P1*vir* | This study |
| MG1655 ∆*dksA ihfA*_FLAG_ | *dksA::tet* allele introduced by transduction using P1*vir* | This study |
| MG1655 ∆*uvrY ihfA*_FLAG_ | *uvrY*_::_*cam* allele introduced by transduction using P1*vir* | This study |
| MG1655 *csrA::kan ihfA*_FLAG_ | *csrA::kan* allele introduced by transduction using P1*vir* | This study |
| MG1655 *ihfB*_FLAG_ | MG1655 with in-frame, CTD 3X –FLAG tag at native *ihfB* locus - *kan*^r^ | This study |
| MG1655 ∆*relA ihfB*_FLAG_ | *relA::kan* allele introduced by transduction using P1*vir* | This study |
| MG1655 ∆*dksA ihfB*_FLAG_ | *dksA::tet* allele introduced by transduction using P1*vir* | This study |
| MG1655 ∆*uvrY ihfB*_FLAG_ | *uvrY*_::_*cam* allele introduced by transduction using P1*vir* | This study |
| MG1655 *csrA::kan ihfB*_FLAG_ | *csrA::kan* allele introduced by transduction using P1*vir* | This study |
| MG1655 *cspA*_FLAG_ | MG1655 with in-frame, CTD 3X –FLAG tag at native *cspA* locus - *kan*^r^ | (3) |
| MG1655 ∆*uvrY cspA*_FLAG_ | *uvrY::cam* allele introduced by transduction using P1*vir* | This study |
| MG1655 *csrA*::*kan cspA*_FLAG_ | *csrA::kan* allele introduced by transduction using P1*vir* | This study |
| MG1655 *csrA*::*kan* ∆*uvrY cspA*_FLAG_ | *uvrY::cam* allele introduced by transduction using P1*vir* in to MG1655 *csrA::kan cspA*_FLAG_ | This study |
| MG1655 *csrA*::*kan* ∆*uvrY* pUY14 *cspA*_FLAG_ | MG1655 *csrA*::*kan* ∆*uvrY cspA*_FLAG_ transformed with pUY14 | This study |
| MG1655 *csrA*::Kan ∆*uvrY pCRA16 cspA*_FLAG_ | MG1655 *csrA*::*kan* ∆*uvrY cspA*_FLAG_ transformed with pCRA16 | This study |
| MG1655 *csrA:*:Kan ∆*uvrY pBR322 cspA*_FLAG_ | MG1655 *csrA*::*kan* ∆*uvrY cspA*_FLAG_ transformed with pBR322 | This study |
| MG1655 *fhuG*_FLAG_ | MG1655 with in-frame, CTD 3X –FLAG tag at native *fhuF* locus - *kan*^r^ | This study |
| MG1655 *uvrY*:: *cam* *fhuF*_FLAG_ | MG1655-*fhuF*_FLAG_ with marked *uvrY* deletion | This study |
| MG1655 *deaD*_FLAG_ | MG1655 with in-frame, NTD 3X –FLAG tag at native *deaD* locus - *kan*^r^ | (3) |
| MG1655 *csrA*:: *kan* *deaD*_FLAG_ | *csrA::kan* allele introduced by transduction using P1*vir* | This study |
| MG1655 *csrA*:: *kan* pCRA16 *deaD*_FLAG_ | MG1655 *csrA*:: *kan* *deaD*_FLAG_ carrying pCRA16 plasmid | This study |
| MG1655 *csrA*:: *kan* pBR322 *deaD*_FLAG_ | MG1655 *csrA*:: *kan* *deaD*_FLAG_ carrying pBR322 plasmid | This study |
| MG1655 *srmB*_FLAG_ | MG1655 with in-frame, CTD 3X –FLAG tag at native *srmB* locus - *kan*^r^ | (3) |
| MG1655 *csrA*::*kan* *srmB*_FLAG_ | *csrA::kan* allele introduced by transduction using P1*vir* | This study |
| CR1-146b | MG1655 *uvrY::kan* with *attλ::P_araB_-araB5’UTR-uvrYFLAG* - *kan*^r^ *tet*^r^ | (3) |
| CR1-146b *csrA*::*kan* | *csrA::kan* allele introduced by transduction using P1*vir* | This study |
| CR1-153 | MG1655 *uvrY::kan* with *attλ::P_araB_-uvrY5’UTR-uvrYFLAG* - *kan*^r^ *tet*^r^ | (3) |
| CR1-153 *csrA*::*gen* | *csrA::gen* allele introduced by transduction using P1*vir* | This study |
| CR1-163 | CF7789 with *attλ::PlacUV5-uvrY12’-’lacZ* - *amp*^r^ | (3) |
| CR1-163 *csrA*::*kan* | *csrA::kan* allele introduced by transduction using P1*vir* | This study |
| CR1-163a | CF7789 with *attλ::PlacUV5-uvrY22’-’lacZ* - *amp*^r^ | (3) |
| CR1-163a *csrA*::*kan* | *csrA::kan* allele introduced by transduction using P1*vir* | This study |
| 14028S | *Salmonella* Typhimurium 14028S | Brian Ahmer |
| 14028S *sirA*FLAG | 14028S with in-frame, CTD *sirA*-FLAG tag at the native *sirA* locus-*kan*^r^ | (3) |
| 14028S *barA*::cam *sirA*FLAG | *Salmonella* Typhimurium 14028S *sirA*_FLAG_ with *barA* deletion | This study |
| 14028S *sirA* | *Salmonella* Typhimurium 14028S with marked *sirA* deletion | Brian Ahmer |
| 14028S *csrB* | *Salmonella* Typhimurium 14028S with marked *csrB* deletion – Cam^r^ | Brian Ahmer |
| BL21_DE3/pET-UvrY | *E. coli* BL21 carrying *uvrY*-HIS6 in a pET24a vector | This study |
| **Plasmids** | **Genotype** | **Source** |
| pKD13 | Construction of Kan-resistant (or markerless) deletions – Kan^r^ Amp^r^ | (4) |
| pKD3 | Construction of *cam*-resistant (or markerless) deletions – *cam*^r^ *amp*^r^ | (4) |
| pKD46 | Lambda Red recombinase expression plasmid | (4) |
| pCP20 | Removal of antibiotic marker from pKD13 or pSUB11 chromosomal replacement – *amp*^r^ *cam*^r^ | (4) |
| pSUB11 | Construction of Kan-resistant (or markerless) CTD 3X-FLAG fusion proteins – *kan*^r^ *amp*^r^ | (5) |
| pET24a | Plasmid used for constructing *uvrY* clone for expressing the UvrY6XHIS protein | Novagen |
| pCRA16 | *csrA* in blunt-ended *Vsp*I site of pBR322, *tet*^r^ | (1) |
| pUY14 | *uvrY* in blunt-ended *Vsp*I site of pBR322, *tet*^r^ | (1) |
| pBR322 | Ectopic expression of proteins – *amp*^r^ *tet*^r^ | (1) |
| pLFX | Vector for constructing transcriptional fusions | (6) |
| pLFXcsrB-lacZ | *csrB* promoter region fused to *lacZ* in pLFX | This study |
| pLFXcsrC-lacZ | *csrC* promoter region fused to *lacZ* in pLFX | This study |
| **Bacteriophages** | **Description** | **Source** |
| Bacteriophage P1 vir | Strictly lytic P1 | Carol Gross |

**Table 2:** Primers used

| **Primer name** | **Sequence 5’🡪 3’** | **Primers used for EMSA** |
| --- | --- | --- |
| *csrB*-ec-MSA-FL-F | gcggaattccttaatcaacttaag | Forward primer used for generating *csrB* DNA probe for electrophoretic mobility shift assay (EMSA). |
| *csrB*-ec-MSA-FL-R | gaagtgtcatcatcctgatgttca | Reverse primer used for generating *csrB* DNA probe for EMSA. |
| *csrC*-ec-MSA-FL-F | gctttgtggcgattatacctgaac | Forward primer used for generating *csrC* DNA probe for EMSA. |
| *csrC*-ec-MSA-FL-R | ctggcgctcctgaccctcatcctg | Reverse primer used for generating *csrC* DNA probe for EMSA. |
| *rrlE*-emsa-ec-nsc-F | caggcaaatccggaaaatcaagg | Forward primer used for generating *rrlE* DNA probe for EMSA. |
| *rrlE*-emsa-ec-nsc(FL)-R | ggcaccattttgcctagttccttc | Reverse primer used for generating *rrlE* DNA probe for EMSA. |
| *cspA*-emsa-ec-F | ttgacgtacagaccattaaagcag | Forward primer used for generating *cspA* DNA probe for EMSA. |
| *cspA*-emsa-ec-R | acgaacacatctttagagccatcg | Reverse primer used for generating *cspA* DNA probe for EMSA. |
| *spF*-emsa-ec-F | ctttctgaactgaacaaaaaagag | Forward primer used for generating *spf* DNA probe for EMSA. |
| *spF*-emsa-ec-R | attactgactggggcggctaaaat | Reverse primer used for generating *spf* DNA probe for EMSA. |
| *fhuF*-emsa-ec-F | tgataatttttatcatttgcaagc | Forward primer used for generating *fhuF* DNA probe for EMSA. |
| *fhuF*-emsa-ec-R | tcatagagcggtgcggaacgatag | Reverse primer used for generating *fhuF* DNA probe for EMSA. |
|  |  | **Primers used for FLAG-tag construction** |
| i*hfA*3XFLAG FWD | gttaaaaagccgggtcgaaaacgcttcgcccaaagacgaggactacaaagaccatgacgg | Forward primer used for constructing C-terminally FLAG-tagged *ihfA.* |
| *ihfA* 3xFLAG reverse | tgtcatggcgttgattttacggtgactcttcgacagtgaacatatgaatatcctccttag | Reverse primer used for constructing C-terminally FLAG-tagged *ihfA.* |
| *ihfA*us 100 | accagccgtacactcgaagaagagg | Forward primer used for confirming the construction of C-terminally FLAG-tagged *ihfA.* |
| *ihfA*ds100 | ggtatccgttctgctgaagtgtcat | Reverse primer used for confirming the construction of C-terminally FLAG-tagged *ihfA.* |
| *ihfB* 3xFLAG fwd | acctggtaaagaactgcgcgatcgcgccaatatttacggtgactacaaagaccatgacgg | Forward primer used for constructing C-terminally FLAG-tagged *ihfB.* |
| *ihfB*3XFLAG rev | cgacaggtgcttttctctcgttcaagtttgagtaaaaaaccatatgaatatcctccttag | Reverse primer used for constructing C-terminally FLAG-tagged *ihfB.* |
| *ihfB* 100us | ggtttcgtcctgtaatcaagcacta | Forward primer used for confirming the construction of C-terminally FLAG-tagged *ihfB.* |
| *ihfB* 100ds | actgttcaaaactctgcgaggcagc | Reverse primer used for confirming the construction of C-terminally FLAG-tagged *ihfB.* |
| *sirA*3XFLG | ccgccatggcctgtgtaatgcggagacgttaacaagccaggactacaaagaccatgacgg | Forward primer used for constructing C-terminally FLAG-tagged *sirA.* |
| *sirA*reverse | gtaacggttttcaaaaacgcctttgcgtcaaatatttcaccatatgaatatcctccttag | Reverse primer used for constructing C-terminally FLAG-tagged *sirA.* |
| ds*sirA*100 | ttaccgacataaataaccgtaccgc | Forward primer used for confirming the construction of C-terminally FLAG-tagged *sirA.* |
| us*sirA*100 | aatacccggtgtttttttacgtttc | Reverse primer used for confirming the construction of C-terminally FLAG-tagged *sirA.* |
|  |  | **Primers used for His6-tag construction** |
| *uvrY*-6xhis-F | cgagttcttcatatgatcaacgttctacttgttgatgaccacgaa | Forward primer used for constructing C-terminally his6-tagged *uvrY.* |
| *uvrY*-6xhis-R | tagctactactcgagctgacttgataatgtctccgcattacacag | Reverse primer used for constructing C-terminally his6-tagged *uvrY.* |
|  |  | **Primers used for gene deletions** |
| *ihfA*-EC-pKD3-F | atggcgcttacaaaagctgaaatgtcagaatatctgtttggtgtaggctggagctgcttc | Forward primer used for *ihfA* deletion. |
| *ihfA*-EC-pKD3-R | ttactcgtctttgggcgaagcgttttcgacccggctttttatgggaa  ttagccatggtcc | Reverse primer used for *ihfA* deletion. |
| *ihfA*-EC-100US-F | accagccgtacactcgaagaagagg | Forward primer used for confirming *ihfA* deletion. |
| *ihfA*-EC-100DS-R | ggtatccgttctgctgaagtgtcat | Reverse primer used for confirming *ihfA* deletion. |
| *cspA*(EC)-pkd13-F | atgtccggtaaaatgactggtatcgtaaaatggttcaacgtgtaggctggagctgcttcg | Forward primer used for *cspA* deletion . |
| *cspA*(EC)-pkd13-R | ttacaggctggttacgttaccagctgccgggcctttagcgctgtcaaacatgagaattaa | Reverse primer used for *cspA* deletion. |
| *cspA(*EC)-100US-F | cgttgatacccctcgtagtgcac | Forward primer used for confirming *cspA* deletion. |
| *cspA*(EC)-100DS-R | cgcgatcgattatttatttcctg | Reverse primer used for confirming *cspA* deletion. |
| *csrB* del-Fwd | gcgccttgtaagacttcgcgaaaaagacgattctatcttcgtgtaggctggagctgcttc | Forward primer used for *csrB* deletion. |
| *csrB* del-Rev | agcaacctcaataagaaaaactgccgcgaaggatagcaggattccggggatccgtcgacc | Reverse primer used for *csrB* deletion. |
| *csrB*-Fwd-PCR | aaggcattgtctgtaagcgccttg | Forward primer used for confirming *csrB* deletion. |
| *csrB-*Rev-PCR | catcgtttcgccagtgctgatgtt | Reverse primer used for confirming *csrB* deletion. |
| *csrC* del-Fwd | gttgattgtttgtttaaagcaaaggcgtaaagtagcacccgtgtaggctggagctgcttc | Forward primer used for *csrC* deletion. |
| *csrC* del-Rev | tcagtatagatttgcggcggaatctaacagaaagcaagcaattccggggatccgtcgacc | Reverse primer used for *csrC* deletion. |
| *csrC*-Fwd-PCR | tgatccagctcctcgcgtgttt | Forward primer used for confirming *csrC* deletion. |
| *csrC*-Rev-PCR | tgtgcaaatactgatggcggttg | Reverse primer used for confirming *csrC* deletion. |
|  |  | **Primers used for Q-RT-PCR** |
| *csrB*-ec-RT-sense | gtaggagatcgccaggaaataag | Q-RT-PCR *csrB* probe set |
| *csrB*-ec-RT-Antisense | agtcttacaaggcgcttacag | Q-RT-PCR *csrB* probe set |
| *lacY*-ec-RT-Sense | ctgctggctggcactattat | Q-RT-PCR *lacY* probe set |
| *lacY*-ec-RT-Antisense | cagcaggaacggtacttcaa | Q-RT-PCR *lacY* probe set |
|  |  | **Primers used for Northern blot** |
| spf- F | taatacgactcactatagggtaaaaaacgttttttaccc | Anti-sense Spf Northern Probe |
| spf- R | gtagggtacagaggtaagatg | Anti-sense Spf Northern Probe |
| csrBR- | gcgttaaaggacacctccagg | Anti-sense CsrB Northern Probe |
| csrBT7 | gtaatacgactcactataggttcgtttcgcagcattccag | Anti-sense CsrB Northern Probe |
| csrCR | gaggacgctaacaggaacaatg | Anti-sense CsrC Northern Probe |
| csrCT7 | gtaatacgactcactataggtcttacaatccttgcaggc | Anti-sense CsrC Northern Probe |
|  |  | **Primers used for ChIP-specificity** |
| *lacY*intrev | gcaaactgttggtcaaaaacatcgt | Forward primer used for amplifying the promoter region of *lacY.* |
| *lacY*intfwd | tggtcgcgcgcggatgtttggc | Reverse primer used for amplifying the promoter region of *lacY.* |
| *csrB*300fwd | gtgcaaagagacgctgggaac | Forward primer used for amplifying the promoter region of *csrB* (*E. coli*). |
| *csrB*300reverse | cctgtcgacgaagatagaatc | Reverse primer used for amplifying the promoter region of *csrB* (*E. coli*). |
| *csrB*300fwd (S) | caatcgtaccgttcttgccgc | Forward primer used for amplifying the promoter region of *csrB* (*S*. Typhimurium). |
| *csrB*300reverse (S) | ttgtacgactccctgtcgacg | Reverse primer used for amplifying the promoter region of *csrB* (*S*. Typhimurium). |
| 16s(S)-F | acgcttgcaccctccgtattac | Forward primer used for amplifying the promoter region of 16S rDNA (*S*. Typhimurium). |
| 16s(S)-R | ctaacacatgcaagtcgaacgg | Reverse primer used for amplifying the promoter region of 16S rDNA (*S*. Typhimurium). |
|  |  | **Primers used for DNase I footprinting** |
| *csrC* DFP Fwd | caggcgcactcatcacaaaatgcgtctg | Forward primer for generating *csrC* template for Foot printing. |
| *csrC* DFP Rev | gtctccggacgtttgtcttcctgac | Reverse primer for generating *csrC* template for Foot printing. |
| *csrB* DFP Fwd | caggaaaatctgattggtcatctggtgac | Forward primer for generating *csrB* template for Foot printing. |
| *csrB* DFP Rev | gtgtcatcatcctgatgttcacttcgttg | Reverse primer for generating *csrB* template for Foot printing. |
|  |  | **Oligonucleotides used for constructing *csrB*-lacZ and *csrC*-lacZ fusions** |
| *csrB lacZ Fwd* | gtcctgcagccggggatatgcacgcgcagtttgt | Forward primer for constructing csrB-lacZ transcriptional fusion |
| *csrB lacZ Rev* | gtcggtaccacgaagatagaatcgtctttttcg | Reverse primer for constructing *csrB*-lacZ transcriptional fusion |
| *csrC lacZ Fwd* | gtcctgcagaatgcgtctgttgataattcaaatta  gtc | Forward primer for constructing *csrC*-lacZ transcriptional fusion |
| *csrC lacZ Rev* | gtcggtacctatgggtgctactttacgcctttgc | Reverse primer for constructing *csrC*-lacZ transcriptional fusion |

REFERENCES CITED

1. Suzuki K, Wang X, Weilbacher T, Pernestig AK, Melefors O, Georgellis D, et al. Regulatory circuitry of the CsrA/CsrB and BarA/UvrY systems of *Escherichia coli*. J Bacteriol. 2002;184(18):5130-40.

2. Romeo T, Gong M, Liu MY, Brun-Zinkernagel AM. Identification and molecular characterization of *csrA*, a pleiotropic gene from *Escherichia coli* that affects glycogen biosynthesis, gluconeogenesis, cell size, and surface properties. J Bacteriol. 1993;175(15):4744-55.

3. Vakulskas CA, Pannuri A, Cortés-Selva D, Zere TR, Ahmer BM, Babitzke P, et al. Global effects of the DEAD-box RNA helicase DeaD (CsdA) on gene expression over a broad range of temperatures. Mol Microbiol. 2014;92(5):945-58.

4. Datsenko KA, Wanner BL. One-step inactivation of chromosomal genes in *Escherichia coli* K-12 using PCR products. Proc Natl Acad Sci U S A. 2000;97(12):6640-5.

5. Uzzau S, Figueroa-Bossi N, Rubino S, Bossi L. Epitope tagging of chromosomal genes in *Salmonella*. Proc Natl Acad Sci U S A. 2001;98(26):15264-9.

6. Edwards AN, Patterson-Fortin LM, Vakulskas CA, Mercante JW, Potrykus K, Vinella D, et al. Circuitry linking the Csr and stringent response global regulatory systems. Mol Microbiol. 2011;80(6):1561-80.
